# Programmable deaminase-free base editors for G-to-Y conversion by engineered glycosylase

**DOI:** 10.1093/nsr/nwad143

**Published:** 2023-05-16

**Authors:** Huawei Tong, Nana Liu, Yinghui Wei, Yingsi Zhou, Yun Li, Danni Wu, Ming Jin, Shuna Cui, Hengbin Li, Guoling Li, Jingxing Zhou, Yuan Yuan, Hainan Zhang, Linyu Shi, Xuan Yao, Hui Yang

**Affiliations:** HuidaGene Therapeutics Co., Ltd., Shanghai 200131, China; HuidaGene Therapeutics Co., Ltd., Shanghai 200131, China; HuidaGene Therapeutics Co., Ltd., Shanghai 200131, China; HuidaGene Therapeutics Co., Ltd., Shanghai 200131, China; HuidaGene Therapeutics Co., Ltd., Shanghai 200131, China; HuidaGene Therapeutics Co., Ltd., Shanghai 200131, China; Department of Neurology and Institute of Neurology of First Affiliated Hospital, Institute of Neuroscience, and Fujian Key Laboratory of Molecular Neurology, Fujian Medical University, Fuzhou 350004, China; HuidaGene Therapeutics Co., Ltd., Shanghai 200131, China; HuidaGene Therapeutics Co., Ltd., Shanghai 200131, China; HuidaGene Therapeutics Co., Ltd., Shanghai 200131, China; HuidaGene Therapeutics Co., Ltd., Shanghai 200131, China; HuidaGene Therapeutics Co., Ltd., Shanghai 200131, China; HuidaGene Therapeutics Co., Ltd., Shanghai 200131, China; HuidaGene Therapeutics Co., Ltd., Shanghai 200131, China; HuidaGene Therapeutics Co., Ltd., Shanghai 200131, China; HuidaGene Therapeutics Co., Ltd., Shanghai 200131, China; Institute of Neuroscience, Center for Excellence in Brain Science and Intelligence Technology, Chinese Academy of Sciences, Shanghai 200031, China; Shanghai Center for Brain Science and Brain-Inspired Intelligence, Shanghai 200031, China

**Keywords:** glycosylase, base excision repair, base editor, depurination, deaminase-free, transversion

## Abstract

Current DNA base editors contain nuclease and DNA deaminase that enables deamination of cytosine (C) or adenine (A), but no method for guanine (G) or thymine (T) editing is available at present. Here we developed a deaminase-free glycosylase-based guanine base editor (gGBE) with G editing ability, by fusing Cas9 nickase with engineered N-methylpurine DNA glycosylase protein (MPG). By several rounds of MPG mutagenesis via unbiased and rational screening using an intron-split EGFP reporter, we demonstrated that gGBE with engineered MPG could increase G editing efficiency by more than 1500 fold. Furthermore, this gGBE exhibited high base editing efficiency (up to 81.2%) and high G-to-T or G-to-C (i.e. G-to-Y) conversion ratio (up to 0.95) in both cultured human cells and mouse embryos. Thus, we have provided a proof-of-concept of a new base editing approach by endowing the engineered DNA glycosylase the capability to selectively excise a new type of substrate.

## INTRODUCTION

**Figure 1.**
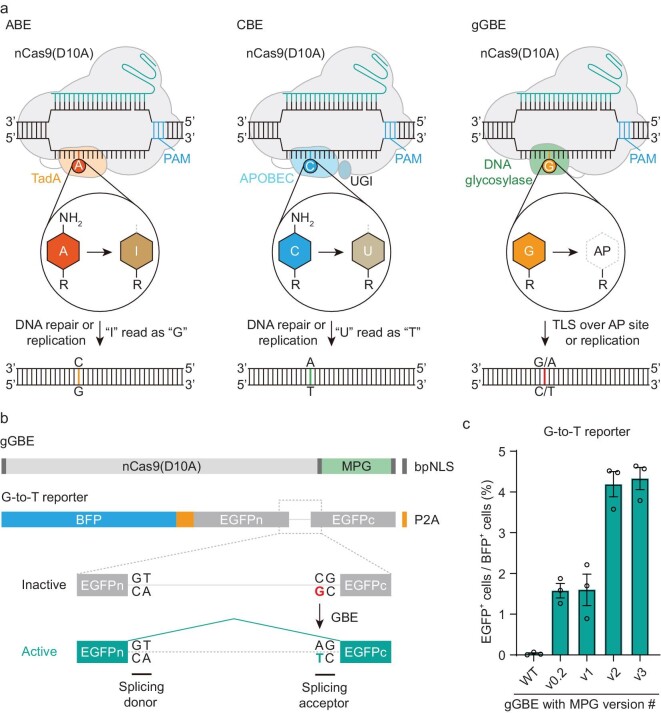
Design and mechanisms of base editing by conventional and glycosylase-based base editors. (a) Schematic diagrams of ABE (left) and CBE (middle) and deaminase-free glycosylase-based guanine base editor (gGBE, right). An nCas9-sgRNA complex creates an R-loop at the target site in the DNA. In ABE and CBE, the evolved adenine deaminase (tRNA adenosine deaminase, TadA) and AID/APOBEC-like cytidine deaminase converts the exposed adenine (A) into deoxyinosine (I) and cytosine (C) into deoxyuridine (U), respectively. In CBE, an additional linked protein, uracil glycosylase inhibitor (UGI), protects U from uracil DNA N-glycosylase (UNG). After deamination, the resulting I is recognized as G, and U as T by DNA polymerase during DNA repair or replication. In gGBE, a prototype version of glycosylase-based guanine base editor (gGBE) designed to remove G, and the AP site generated is repaired by TLS and/or DNA replication, leading to G-to-C or G-to-T conversion. PAM, protospacer adjacent motif. AP, apurinic/apyrimidinic sites. (b) A screening reporter system for detecting G-to-T conversion by gGBE. P2A, 2A peptide from porcine teschovirus-1. (c) Percentage of EGFP^+^ cells for G editing activity evaluation of gGBE with various MPG variants (mean ± s.e.m., *n* = 3). WT: wild-type.

Base editing is a powerful technology for basic research and therapeutic applications [1,2]. Current base editors mainly contain programmable DNA-binding proteins, such as a catalytically impaired CRISPR-associated (Cas) nuclease that was fused with a single-stranded DNA deaminase enzyme and sometimes an additional protein that could modulate DNA repair machinery [3,4]. In the past several years, two main classes of DNA base editors, adenine base editors (ABEs) [5] and cytosine base editors (CBEs) [4] have been developed and widely used for A-to-G and C-to-T conversions, respectively (Fig. 1a). Recently, C-to-G base editors (CGBEs) [6–10] and adenine transversion base editor (AYBE) [11] were also constructed by fusing existing CBE or ABE with DNA glycosylase variants to generate new tools for achieving more versatile base editing outcomes, including C-to-G, A-to-C and A-to-T editing (Supplementary Fig. S1). CRISPR-free CBEs (DdCBEs) were reported for performing C-to-T base editing in mitochondria DNA, by fusing two halves of a double-strand DNA cytidine deaminase (DddA) variants with two separate TALE (transcription activator-like effector) proteins [12–14]. So far, these base editing methods all began with deamination of C and A as the key step to produce deoxyuridine (U) and deoxyinosine (I) intermediates, respectively, which in turn were transformed into other bases by endogenous DNA repair or replication mechanisms [4–11]. Although G and T in the non-edited strand could be modified along with the event of editing of C and A in the edited strand, respectively, no existing base editor is capable of directly editing G or T.

Base excision proteins in base excision repair (BER) pathways have been successfully harnessed for developing new base editing tools [4–11,15]. The mechanism of BER involves a damage-specific step performed by one of the DNA glycosylases, followed by base lesion events, most frequently spontaneous DNA depurination and deamination [16,17]. Mutagenesis by deamination is unlikely for G (due to spontaneous remediation) [18] and impossible for T (due to the absence of amine), making the development of G and T base editors a challenging task. Depurination of damaged G and A occurs frequently in genomic DNA and generates apurinic/apyrimidinic (AP) sites by DNA glycosylases, initiating BER that readily remediates the loss of these bases [19,20].

There are many types of DNA glycosylases, including N-methylpurine DNA glycosylase (MPG), 8-oxoguanine DNA glycosylase (OGG1) and mutY DNA glycosylase (MUTYH), that could excise various damaged purine bases [19,21–23]. The MPG (also known as alkyladenine DNA glycosylase, AAG) is one of the first responders to DNA base damage and capable of recognizing a wide range of damaged purine bases, including alkylpurines like 3-methyladenine (3meA) and 7-methylguanine (7meG), oxidized adenine 1, N^6^-ethenoadenine (ϵA) and deaminated adenine hypoxanthine (Hx) [24–29]. Although MPG may also remove normal G at very low rates *in vitro* [30], overexpression of MPG in transgenic mice did not result in additional point mutations as compared to that found in WT mice, supporting the notion that AP sites due to MPG excision are quickly processed by apurinic/apyrimidinic endonuclease 1 (APE1) into single-strand breaks (SSBs), which could not be mutated by translesion synthesis (TLS) [31]. This implies that depurination-initiated BER could be used for developing novel base editing tools that may prove to be reliable and free of side effects. We speculated that excision of canonical G could be achieved by engineering the MPG protein moiety, leading to efficient base editing through the BER pathway.

Here, we developed a novel tool, namely glycosylase-based guanine base editor (gGBE), to achieve high editing efficiency for G-to-C and G-to-T conversion in mammalian cells. After four rounds of mutagenesis, we obtained marked enhancement of G base editing activity, as compared with that obtained by using wild-type (WT) MPG. We characterized the editing profile of gGBE by targeting dozens of endogenous genomic loci in cultured mammalian cells as well as mouse embryos, demonstrating its high G-to-Y (Y = C or T) base editing efficiency. Thus, DNA glycosylase could be engineered into a new base editing tool.

## RESULTS

### Development of glycosylase-based base editors

We have attempted to develop a novel base editing system by harnessing the BER pathway triggered by depurination of G or A. By fusing human MPG at the C-terminus of Cas9 D10A nickase (nCas9), we generated a prototype version of deaminase-free glycosylase-based base editor (gBE), in which MPG may remove normal G or A, two purines with similar structure of Hx, to generate AP sites, with nCas9 nicking the opposite non-edited strand. This allowed the damaged DNA to preferentially use the edited strand as a template for DNA repair and/or DNA replication. Therefore, nucleotide incorporation opposite the AP site via TLS would lead to diverse editing outcomes (Fig. 1a). To conveniently evaluate the gBE editing activity, we developed a simple intron-split EGFP reporter system as previously reported [11]. Disruptive point mutations (AG to CG or TG) were introduced in the intron boundary to generate inactive splicing acceptor (SA) signals, and G-to-T or A-to-T conversion (C-to-A or T-to-A conversion in the opposite strand) was required to correct the mutation for proper splicing of the EGFP-coding sequence, thus activating EGFP expression (Fig. 1b and Supplementary Fig. S2a). The EGFP fluorescence intensity could be detected with flow cytometry (Supplementary Fig. S2d). The G-to-T reporter is to be used for evaluating the activity of glycosylase-based guanine base editor (gGBE), and the A-to-T reporter for evaluating the glycosylase-based adenine base editor (gABE). We first constructed gBE vectors with different versions of MPG (MPGv0.2 to MPGv3) that were reported in our previous study [11]. After co-transfecting the G-to-T or A-to-T reporter vector with the gBE vector containing the single-guide RNA (sgRNA) that targets the intronic mis-splicing mutation, we found that all the gBE showed G-to-T but not A-to-T conversion activity (Fig. 1c and Supplementary Fig. S2b). The differences between normal G and A substrate recognition by MPG might account for the failure of A editing in this system. Among them, gBE containing MPGv3 (hereafter referred to as gGBEv3, carrying G163R, N169S, S198A, K202A, G203A, S206A and K210A mutations) exhibited the highest G-to-T editing activity (4.33%) in cultured HEK293T cells (Fig. 1c) as compared to that of gGBE with WT MPG (0.03%), a striking 144-fold enhancement in the editing efficiency. Furthermore, no conversion activity was found for gGBE with dead MPG (dMPG, carrying E125A, Y127A and H136A mutations) or gGBEv3 together with non-targeting sgRNA (Supplementary Fig. S2c). Thus, the G-to-T conversion by gGBEv3 depends on the catalytic G excision domain of MPG and specific sgRNA.

### Further enhancement of G editing activity of gGBE

**Figure 2.**
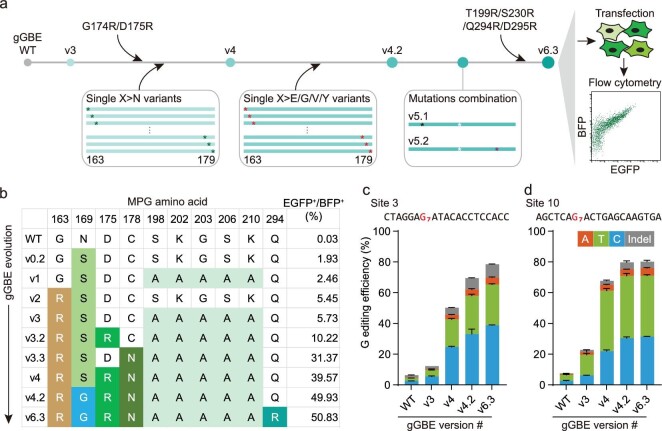
Mutagenesis of the MPG moiety in gGBEs. (a) Schematic diagram of mutagenesis and screening strategy for the engineered gGBE. The EGFP reporter plasmids were transiently co-transfected into cultured cells along with the gGBE plasmids. (b) Genotypes of a subset of engineered gGBEs, with percentage of EGFP^+^ cells for each gGBE variant on the far-right column (more engineered variants listed in Supplementary Fig. S5). Different steps of mutagenesis (see text) are marked by different shaded colors. (c and d) G base editing outcomes with different gGBE variants at the edited G7 position in site 3 (c) and site 10 (d) in transfected HEK293T cells by target deep sequencing (mean ± s.e.m., *n* = 3).

To further increase the G-to-T activity of gGBEv3, we performed protein engineering by several rounds of rational mutagenesis of the MPG moiety, using the G-to-T reporter to evaluate the editing activity (Fig. 2a). Based on structural analysis of MPG [32,33], we selected the 17-aa-long region from R163 to V179 (R163–V179) of MPGv3, which forms a pocket around the targeted G base in a model of MPG-DNA complex (Supplementary Fig. S3a). First, we mutated gGBEv3 at G174R or D175R, two mutations known to improve AYBE activity [11], and generated two variants gGBEv3.1 (v3 with G174R) and gGBEv3.2 (v3 with D175R). We found that the G-to-T conversion activity for gGBEv3.2 was 1.78-fold of that found for gGBEv3 (Fig. 2a and Supplementary Fig. S3b). We also attempted to scan the R163–V179 region with sequential substitutions of asparagine (X > N), which is critical for substrate recognition by MPG [29,34]. Interestingly, we obtained a variant gGBEv3.3 (v3 with C178N), which substantially elevated the G-to-T conversion activity by 5.5-fold (Fig. 2a and Supplementary Fig. S3c). Furthermore, in a variant gGBEv4 (v3 with both D175R and C178N), we found a synergistic enhancement of G-to-T editing activity by 6.9-fold in comparison with the G editing activity of gGBEv3 (Fig. 2a and Supplementary Fig. S3b). In addition, the gGBEv4 with MPG fused at the C-terminus showed slightly higher editing activity than that at the N-terminus (34.6% vs. 25.9%, Supplementary Fig. S3d).

We then performed another round of mutagenesis and screening based on gGBEv4 to further elevate G editing activity of gGBE. We mutated the R163–V179 region of MPGv4 by sequential replacement with amino acids of distinct properties, including glutamic acid (with negative charged side chain), valine (with small hydrophobic side chain), glycine (with no side chain), or tyrosine (with large hydrophobic side chain) (X > E, V, G or Y). Although most of these mutations reduced the G editing activity, we found three variants, gGBEv4.1 (v4 with I170V), gGBEv4.2 (v4 with S169G) and gGBEv4.3 (v4 with R163Y), that showed elevated editing efficiency by 1.06-, 1.28- and 1.09-fold, respectively (Supplementary Fig. S4), as compared with that of gGBEv4. We also attempted to generate two additional variants (gGBEv5.1 and gGBEv5.2) by combining the above three effective mutations, but found no further enhancement (Supplementary Fig. S5). Encouraged by the above finding on the variant gGBEv3.2, we further introduced T199R, S230R, Q294R or D295R individually into gGBEv4.2, mutations previously found to improve conversion activity of AYBE [11]. We found that gGBEv6.3 (v4.2 with Q294R) further increased the editing efficiency by 1.02- and 1694-folds, as compared with gGBEv4.2 and gGBE with WT MPG, respectively (Fig. 2b and Supplementary Fig. S5).

We next validated the enhancement of G editing activity of gGBE variants obtained above at two endogenous genomic sites in cultured HEK293T cells. The cells were transfected with a construct encoding each gGBE variant, together with mCherry and sgRNA that targeted site 3 (or site 10), and mCherry^+^ cells were FACS-sorted for target deep sequencing analysis. We obtained a gradual elevation of overall G editing efficiency at G7 from 6.4% to 78.5% for site 3, and from 7.5% to 80.3% for site 10, respectively (Fig. 2c and d). The G-to-C and G-to-T conversions were the predominant events at these two sites, and only a low percentage of G-to-A conversion (4.6% for site 3, 5.0% for site 10) and a few insertions and deletions (indels, 8.5% for site 3; 4.3% for site 10) were detected for gGBEv6.3. These results indicate that the four rounds of mutagenesis described above had effectively optimized gGBE activity for G-to-C and G-to-T base editing. Taken together, the engineered version of gGBEv6.3 (carrying G163R, N169G, D175R, C178N, S198A, K202A, G203A, S206A, K210A, Q294R mutations) had the highest G editing efficiency and was used for the following studies.

### Characterization of gGBEv6.3 at human genomic DNA sites

**Figure 3.**
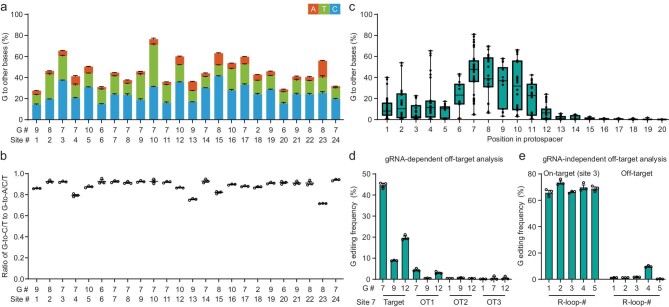
Characterization of editing profiles of gGBE via target deep sequencing. (a) Bar plots showing the on-target DNA base editing at positions with the highest G conversion frequencies at each genomic site in HEK293T cells (mean ± s.e.m., *n* = 3). G#: G position with highest on-target base editing frequencies across protospacer positions 1–20; site #: genomic site number. (b) The ratio of G-to-C/T to G-to-A/C/T conversion frequency by gGBEv6.3 editing at the sites shown in **a**. (c) Frequencies of G conversions by gGBEv6.3 across protospacer positions 1–20 at the edited sites in **a** (in which PAM was at positions 21–23). Single dots represent individual data point from three independent replicates per site. Boxes span the interquartile range (25th to 75th percentile); horizontal line in the box indicates the median (50th percentile); small horizontal bars mark the minimal and maximal values. (d) The sgRNA-dependent off-target analysis for gGBEv6.3 editing efficiency at site 7 (mean ± s.e.m, *n* = 3). OT: off-target. (e) The sgRNA-independent off-target editing efficiency detected by the orthogonal R-loop assay at each R-loop site (mean ± s.e.m, *n* = 3).

We further characterized the editing profiles of gGBEv6.3 by targeting 24 endogenous genomic loci, most of which were used in previous base editing studies [9,35,36]. We found that gGBEv6.3 achieved efficient G base editing activity (ranging from 27.6% to 76.5%), with predominately G-to-C and G-to-T conversions, but essentially no A, C or T editing at all 24 sites examined (Fig. 3a, c and Supplementary Fig. S6a–c). Among all cells examined, the ratio of G-to-C/T (G-to-Y, Y = C and T) to G-to-A/T/C conversion was high (ranging from 0.72 to 0.94, Fig. 3b). Only a low percentage of G-to-A conversion was detected (Fig. 3a and Supplementary Fig. S6e–g), consistent with previous results with AYBE [11] and CGBEs [6–10]. We found that gGBEv6.3 also induced indel at the 24 edited sites with frequency ranging from 4.7% to 30.0% (Supplementary Fig. S6h). Furthermore, we found that the editable range of gGBEv6.3 was positions 1 to 14, and the optimal editing window with high efficiency of G conversion covered protospacer positions 6 to 11, with the highest editing efficiency at position 7 (Fig. 3c, Supplementary Fig. S6d and i). The analysis of on-target editing and sequences of all the sites showed that gGBEv6.3 performed no obvious NG motif preference for G conversions (Supplementary Fig. S6j).

We have analyzed the guide-dependent off-target activity of gGBEv6.3 at several previously reported [11,35] and in silico-predicted [37] guide-dependent off-target sites, and characterized the ability of gGBEv6.3 to mediate guide-*independent* off-target DNA editing using orthogonal R-loop assay in five dSaCas9 R-loops, reported in previous studies [11,35]. We found similar or lower percentage of editing at the guide-dependent off-target loci (Fig. 3d and Supplementary Fig. S7a), as compared with that of adenine base editors found previously [11,35]. Moreover, among five guide-independent off-target sites, we detected very low frequencies (1.1% on average) at four sites (Fig. 3e and Supplementary Fig. S7b), and a slightly higher frequency in one site, which harbored 12 Gs across the entire protospacer. Taken together, we found that gGBEv6.3 represents a highly efficient G-to-Y base editor with a low off-target effect in mammalian cells.

### Potential gene-editing applications of gGBE

**Figure 4.**
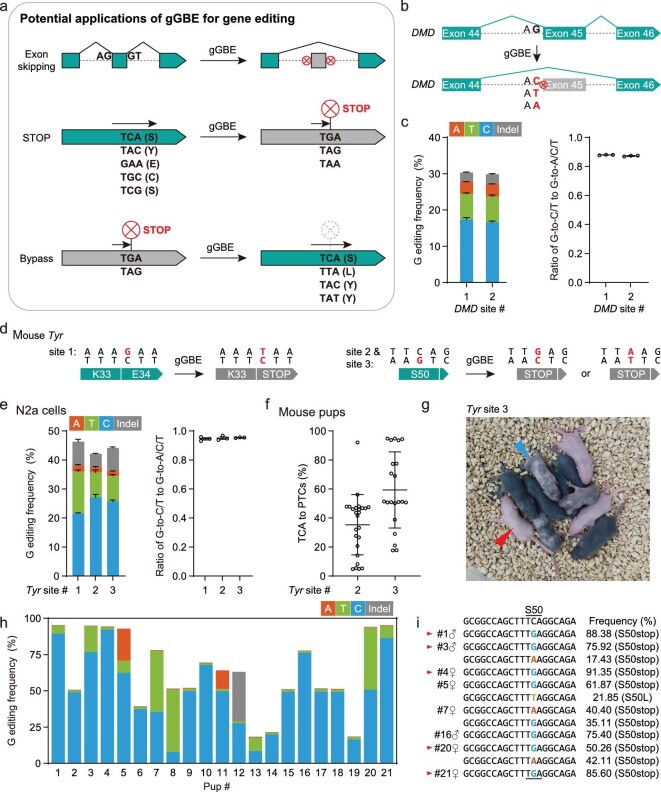
Gene editing applications of gGBE. (a) Application of gGBEv6.3 for editing splicing sites, introduction of premature termination codons (PTCs), as well as editing that bypasses PTCs. (b) Schematic diagram illustrating gGBE-indued skipping of *DMD* exon 45. (c) Bar plots showing the on-target DNA base editing frequencies of G editing and the ratio of G-to-C/T to G-to-A/C/T editing frequencies by gGBEv6.3 at two *DMD* sites in HEK293T cells (mean ± s.e.m, *n* = 3). SpG, a PAM-flexible Cas9 variant, was used for targeting *DMD* site 2. (d) Schematic diagram illustrating the introduction of PTCs in the mouse *Tyr* gene by gGBE. (e) Bar plots showing the on-target G editing frequencies and G-to-Y Ratio by gGBEv6.3 at three *Tyr* sites in N2a cultured cells (mean ± s.e.m, *n* = 3). (f) Percentages of G conversion-induced PTCs by gGBEv6.3 at two *Tyr* sites in mouse pups (mean ± s.e.m., *n* = 25 and 21 pups for site 2 and site 3, respectively). (g) Phenotype of F0 mice generated by gGBE editing in mouse zygotes. The image showing the presence of edited P6 mice. Red arrowhead, albino; blue arrowhead, mosaic pigmentation. (h) Bar plots showing the on-target G editing frequencies for individual mouse pups, with gGBEv6.3 targeting *Tyr* site 3. (i) Genotyping of representative F0 pups from (h). The frequencies of mutant alleles were determined by high-throughput sequencing. Red arrowhead, albino pups.

The G-to-Y conversion ability of gGBE allows for a variety of gene-editing applications, including editing splicing sites, introduction of premature termination codons (PTCs), as well as editing that bypasses PTCs (Fig. 4a). The inactive splicing acceptor (SA) signal with disruptive point mutations, exemplified by intron-split EGFP reporter system used above, could be remediated with gGBE (Fig. 1b). Conversely, gGBE could be used for disrupting the splicing signal by converting G within a splicing donor site (‘GT’) or splicing acceptor site (‘AG’) to other bases, resulting in exon skipping. To illustrate this application, we targeted the splice acceptor site of *DMD* (Duchenne muscular dystrophy) exon 45 with gGBEv6.3, and achieved a high efficiency of G editing (up to 30.3%) with a high G-to-Y ratio (up to 0.88) when targeting *DMD* site 1 (Fig. 4b, c and Supplementary Fig. S8a).

Another application of gGBE is to introduce PTCs to disrupt gene expression by converting TCA, TAC and GAA codons into stop codons TGA, TAG or TAA. Note that PTC by GAA to TAA conversion could be introduced only by using gGBEv6.3, no other current base editor could induce this type of PTC. By targeting three sites in the mouse *Tyr* (*Tyrosinase*, associated with coat color) gene with gGBEv6.3 for creating PTCs (Fig. 4d) in cultured N2a cells, we achieved a high efficiency of G editing (up to 46.3%) with high G-to-Y ratio (up to 0.95) (Fig. 4e and Supplementary Fig. S8b). To further illustrate the potential *in vivo* application, we co-injected gGBEv6.3 mRNA and *Tyr*-targeting sgRNA into mouse zygotes of C57BL6 background (black coat color), with 20 mouse embryos used for each of three *Tyr*-targeting sgRNAs. We found highly efficient G editing (Supplementary Fig. S9a) for two of three sgRNAs, with an average of 50.9% PTC introduction efficiency when targeting the *Tyr* site 3 (Supplementary Fig. S9b and c). Similar to the data obtained above in cultured cell lines (Fig. 4e), gGBE induced very few indels in mouse embryos (Supplementary Fig. S9d and e). When targeting the *Tyr* site 3 with gGBEv6.3, all the 21 F0 pups had G conversions with an average of 59.4% efficiency (Fig. 4f–h). This gGBEv6.3-induced G-to-Y editing resulted in the albino or mosaic phenotype in the coat color of F0 mice (Supplementary Fig. S10), suggesting efficient disruption of the tyrosinase activity (Fig. 4i). Thus, gGBEv6.3 was an efficient G base editor not only in cell lines but also in mouse embryos.

## DISCUSSION

Two major classes of deaminase-based base editors (dBE), ABE and CBE, as well as their derivatives (such as AYBE and CGBE), perform base editing with deamination of A or C as the first key step [3–11]. In this study, we designed deaminase-free editors based on engineered MPG, and generated a gGBE editor that could achieve highly efficient G-to-C and G-to-T conversion in both cultured human cells and mouse embryos. This high editing efficiency could be attributed to mutations in the MPG protein that facilitate its specific substrate selection or DNA-binding activity, or both, which needs to be elucidated by structural and biochemical experiments in the future.

In all human pathogenic SNPs (60 372 total), there are about 10% C-to-G and 5% T-to-G SNPs [11]. Although C-to-G SNPs could be corrected by CGBE [6–10], we could not usually find an efficient sgRNA due to the PAM limitation and narrow editing window. The gGBE could increase the opportunity to find an efficient sgRNA by targeting the opposite strand compared with CGBE. For T-to-G SNPs, no current base editor could efficiently induce G-to-T (or C-to-A in the opposite strand) conversion. Therefore, gGBE greatly broadens the targeting scope of base editors.

Although we have found a low off-target effect of the gGBE editor on several targeted genes in culture mammalian cells, a comprehensive evaluation of gGBEs through high-throughput whole-genome sequencing methods, such as GOTI [38,39], SAFETI [40] and Detect-seq [41], is required for a thorough assessment of off-target effects. Although the editable range (positions 1 to 14) is wide with the current version of gGBE (Fig. 3c), a more accurate gGBE with a refined editing window, like ABE9 [42] or YE1-BE3 [43], might be achieved through further engineering of the glycosylase moiety or architectures of gGBE. Moreover, further structural fine-tuning of gGBE could potentially achieve more specific G-to-C and G-to-T editors by co-expression of deaminases or modifying DNA repair machinery (e.g. co-expression of a specific TLS polymerase) [11,44,45]. We note that indels generated by gGBE, as well as by AYBE and CGBEs that created AP sites, were several folds higher than those generated by ABE or CBE [3–11]. Thus, additional effort is required to reduce the indel frequency. Encouraged by the previous studies on CGBE [7,10] and AYBE [11], the level of off-target editing or indels could be reduced through protein or sgRNA engineering approaches in the future.

In summary, we have shown that DNA glycosylases could be engineered into proteins that selectively excise a specific nucleotide base. Thus, besides G base editing illustrated here, glycosylase-based base editor could also be developed by similar protein engineering methods for base editing of A, C and T, providing a complete set of base editing tools for gene editing studies.

## MATERIALS AND METHODS

### Molecular cloning

Base editor constructs used in this study were cloned into a mammalian expression plasmid backbone under the control of a EF1α promoter by standard molecular cloning techniques. Intron-split EGFP reporters were engineered as previously described [11]. In brief, corresponding mutations at the splice acceptor site were made to construct A-to-T reporter or G-to-T reporter via site-directed mutagenesis by PCR, respectively. Mutations at the splice acceptor site led to inactive EGFP production by non-spliced EGFP transcripts. Encouraged by the findings from previous base editors [7,10], modification of the 68th base (G-to-C) was made in the intron sequence for introducing artificial PAM on the template strand, thus the corresponding mutations at the splice acceptor site were at position 6 across the protospacer. Transversion corrections in A-to-T reporter or G-to-T reporter were required for proper splicing of EGFP-coding sequence. MPG mutagenesis libraries were designed and generated as previously described [46]. The 17aa-long region from R163 to V179 (R163–V179) of MPGv3 was selected for protein engineering. BpiI-harboring mutant, MPG- G174R/D175R/T199R/S230R/Q294R/D295R mutants or corresponding combinations were constructed via site-directed mutagenesis by PCR. Sequential asparagine/glutamic acid/valine/glycine/tyrosine substitutions (X > N, E, V, G or Y) were designed, with oligos coding for the mutants annealed and ligated into corresponding BpiI-digested backbone vectors. The gRNA oligos were annealed and ligated into BpiI sites. The amino-acid sequence for gGBEv6.3 is supplied in Supplementary Table 1. The MPG mutants and corresponding codon substitutions used are listed in Supplementary Table 2.

### Analysis of target sequencing data

Target sequencing data analysis has been previously described [11]. In brief, the targeted amplicon sequencing reads were processed using fastp with default parameters [47]. The cleaned pairs were then merged using FLASH v1.2.11. The amplified sequences from individual targets were demultiplexed using fastx_barcode_splitter.pl from fastx_toolkit (0.0.14). Further amplicon sequencing analysis was performed by CRISPResso2 [48]. A 10-bp window was used to quantify modifications centered around the middle of the 20-bp gRNA. Otherwise, the default parameters were used for analysis. G-to-C purity was calculated as G-to-C editing efficiency/(G-to-C editing efficiency + G-to-T editing efficiency + G-to-A editing efficiency). G-to-Y conversion ratio was calculated as (G-to-C editing efficiency + G-to-T editing efficiency)/(G-to-C editing efficiency + G-to-T editing efficiency + G-to-A editing efficiency).

## DATA AVAILABILITY

Expression plasmids used in this study have been deposited at Addgene and will be available at https://www.addgene.org/Huawei_Tong/ (Addgene plasmid nos. 202629-202630). All data are available in the main text or the Supplementary Materials. Targeted amplicon sequencing data have been deposited at the Sequence Read Archive and can be accessed at https://www.ncbi.nlm.nih.gov/bioproject/PRJNA971099. All relevant original data are available from the corresponding authors upon request.

## Supplementary Material

nwad143_Supplemental_Files
