# Supplementary material for: Programmable deaminase-free base editors for G-to-Y conversion by engineered glycosylase
Source: Natl Sci Rev. 2023 May 16;10(8):nwad143. doi: 10.1093/nsr/nwad143 (PMC10317176; doi:10.1093/nsr/nwad143)
Supplement: nwad143_Supplemental_Files [file nwad143_supplemental_files.zip › 20230513_NSR_gGBE_supp.docx]

**SUPPLEMENTARY DATA**

**SUPPLEMENTARY MATERIALS AND METHODS**

**Cell culture, Transfection, and flow cytometry analysis**

HEK293T cells were cultured with DMEM (Catalog# 11995065, Gibco) supplemented with 10% fetal bovine serum (Catalog# 04-001-1ACS, BI) and 0.1 mM non-essential amino acids (Catalog# 11140-050, Gibco). Cells were grown in an incubator at 37 °C with 5% CO_2_. MPG mutant screening was conducted in 48-well plates. The day before transfection, 3 × 10^4^ HEK293T cells per well were plated in 250 μL of complete growth medium in the 48-well plates. After 12h, 500 ng gGBE plasmids and 500 ng A-to-T or G-to-T reporter plasmids were co-transfected into cells with 2 μg Polyethylenimine (PEI) (DNA/PEI ratio of 1:2) per well. For cell transfection of HEK293T for FACS, 5 × 10^5^ cells per well were plated in 12-well plates with 1 ml complete growth medium the day before transfection. After 14-16h, 2 μg gGBE-sgRNA plasmids were transfected into cells using PEI (DNA/PEI ratio of 1:2). For targeting *DMD* site 2 with an NG PAM, a PAM-flexible Cas9 variant SpG was used. Orthogonal R-loop assays were performed as described previously [1, 2]. In brief, 1 μg of gGBE plasmid with sgRNA targeting site 3 and 1 μg of dSaCas9 plasmid with corresponding sgRNA targeting five off-target sites to generate R-loops were co-transfected into HEK293T cells in 12-well plates using PEI (DNA/PEI ratio of 1:2). 48h after transfection, expression of mCherry, BFP and EGFP fluorescence were analyzed by BD FACS Aria III or Beckman CytoFLEX S. Flow cytometry results were analyzed with FlowJo V10.5.3. The gating strategy in the identification of mCherry^+^, BFP^+^ and EGFP^+^ cells for on-target editing efficiency evaluation was supplied in Supplementary Fig. S2d.

**Animals**

Experiments involving mice were approved by the Biomedical Research Ethics Committee of Center for HuidaGene Therapeutics Co. Ltd. Super ovulated C57BL/6 females (4 weeks old) were mated with C57BL/6 males (8 weeks old), and females from the ICR strain were used as foster mothers. Mice were maintained in a specific pathogen-free facility under a 12-hour dark–light cycle, and constant temperature (20–26°C) and humidity (40–60%) maintenance.

**In *vitro* transcription of gGBE mRNA and *Tyr*-sgRNAs**

The gGBE plasmids were structured by standard PCR amplification with Phanta Max Super-Fidelity DNA Polymerase (Vazyme Biotech Co., Ltd), assembly with Gibson Assembly Master Mix (NEB E2611L), and transformation into chemically competent DH5α cells. The gGBE plasmids were linearized by the FastDigest KpnI restriction enzyme (Thermo Fisher), purified using Gel Extraction Kit (Omega), and used as the template for *in vitro* transcription (IVT) using the mMESSAGE mMACHINE T7 Ultra kit (Life Technologies). For *Tyr*-sgRNAs preparation, we added the T7 promoter sequence to the sgRNA template by PCR amplification of px330 (Addgene, #42230) using the primer pair listed in Supplementary Table xx. The T7-*Tyr*-sgRNA PCR product was purified using Gel Extraction Kit (Omega) and used as the template for IVT of sgRNAs using the MEGAshortscript T7 kit (Life Technologies). The gGBE mRNA and *Tyr*-sgRNAs were purified using the MEGAclear kit (Life Technologies) and eluted in RNase-free water. *In vitro* transcribed RNAs were aliquoted and stored at -80°C until use. Prior to microinjection, the mixture of gGBE mRNA and *Tyr*-sgRNA was prepared by centrifuge for 10 min at 14,000 rpm at 4°C and supernatant transferred to 0.2 mL fresh PCR tubes for injection.

**Microinjection of mouse zygotes with gGBE mRNA and *Tyr*-sgRNA**

Super-ovulated C57BL/6 females (4 weeks old) were mated with C57BL/6 males, and fertilized embryos were collected from oviducts 21 h post hCG injection. For zygote injection, the mixture of gGBE mRNA (100 ng/µL) and Tyr-sgRNAs (100 ng/µL) was injected into the cytoplasm of 1-cell embryo in a droplet of M2 medium using a FemtoJet microinjector (Eppendorf) with constant flow settings. The injected embryos were cultured in M16 medium with amino acids at 37°C under 5% CO2 in air for 2 hours and then transferred into oviducts of pseudo-pregnant ICR foster mothers at 0.5-d.p.c.

**Target sequencing of endogenous sites**

At 72 h post-transfection, 10,000 mCherry positive cells were isolated by FACS. Genomic DNA was extracted by addition of 40 μl of lysis buffer and 1 μL Proteinase K (Catalog# PD101-01, Vazyme) directly into each tube of sorted cells. The genomic DNA/lysis buffer mixture was incubated at 55 °C for 45 min, followed by a 95 °C enzyme inactivation step for 10 min. The regions of interest for target sites were amplified by PCR using site-specific primers. The PCR reaction was performed at 95 °C for 5 min, 30 cycles at 95 °C for 15 s, 60 °C for 15 s, 72 °C 30 s, and a final extension at 72 °C for 5 min using Phanta ® Max Super-Fidelity DNA Polymerase (Catalog# P505-d3, Vazyme). PCR products were purified using universal DNA purification kit (TIANGEN) according to the manufacturer’s instructions, and analyzed by Sanger sequencing (Genewiz). The amplicons were ligated to adapters and sequencing was performed on the Illumina MiSeq platforms. Protospacer sequences and site-specific primers used for each genomic locus are listed in Supplementary Table 3 and 4.

**Statistical analysis**

Statistical tests performed by Graphpad Prism 8 included the two-tailed unpaired two-sample *t*-test or Dunnett's multiple comparisons test after one-way ANOVA.

**SUPPLEMENTARY FIGURES**


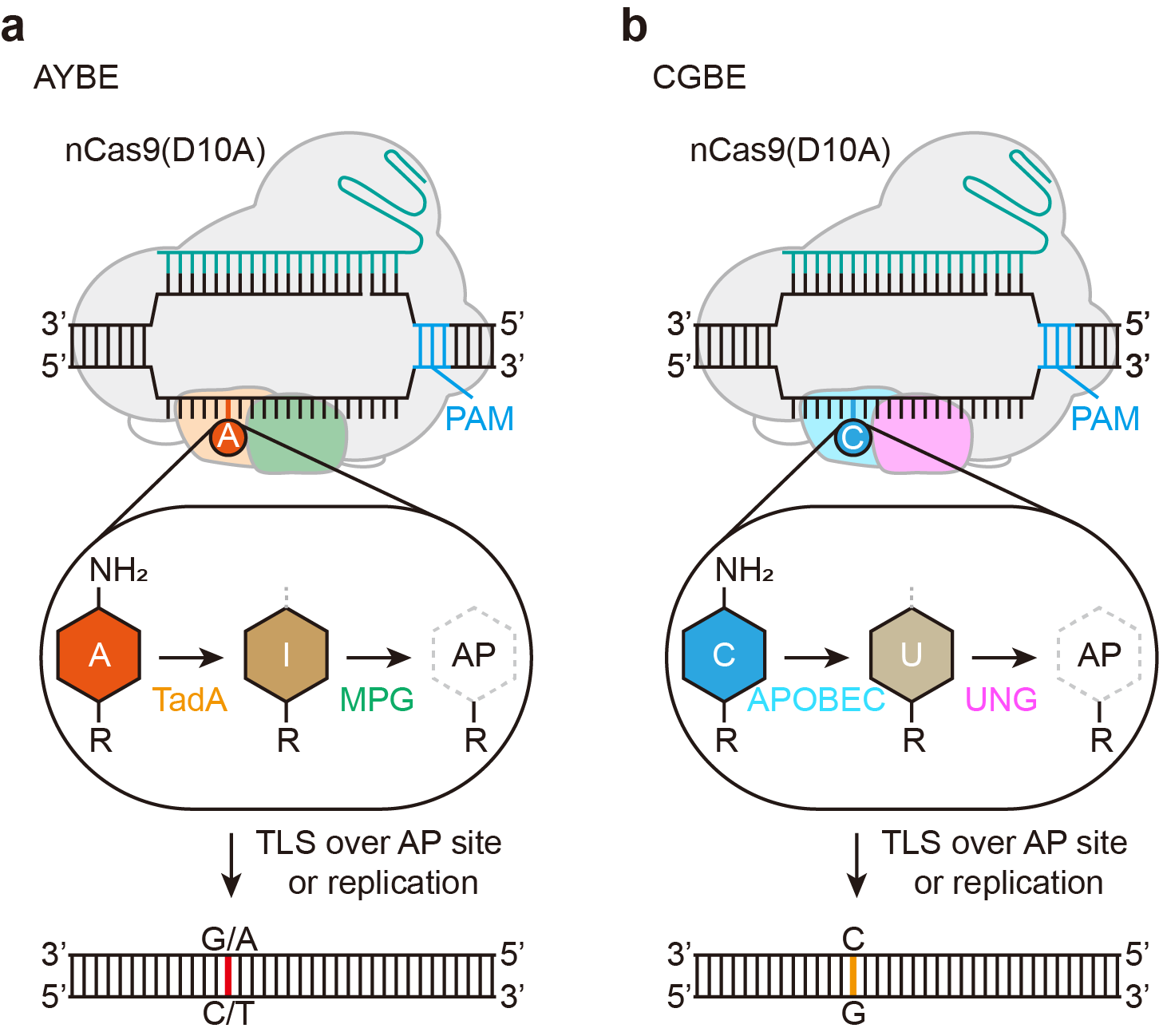


**Supplementary Figure 1.** Overview of AYBE and CGBE. (a) Schematic diagram of AYBE. N-methylpurine DNA glycosylase (MPG) excises the resulting inosine (I) from deamination of adenine (A) by the adenine deaminase (TadA), triggering the base excision repair (BER) pathway in cells, thus causing more versatile base editing outcomes, including A-to-C and A-to-T editing. (b) Schematic diagram of CGBE. Uracil DNA N-glycosylase (UNG) excises the resulting uridine (U) from deamination of cytosine (C) by the AID/APOBEC-like cytidine deaminase, triggering the base excision repair (BER) pathway in cells, thus causing dominant C-to-G editing. PAM, Protospacer adjacent motif. AP, apurinic/apyrimidinic sites.


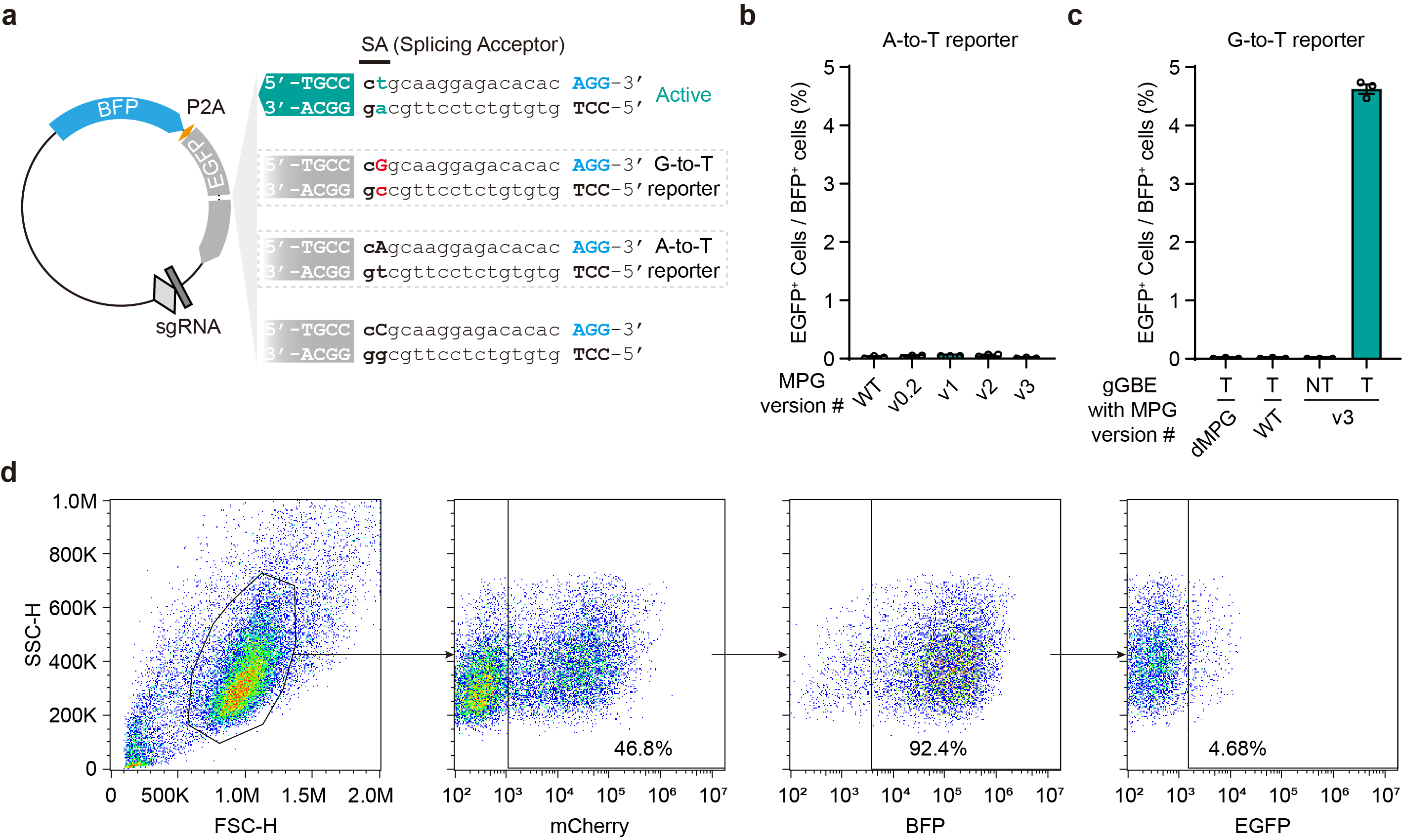


**Supplementary Figure 2.** Characterization of A-to-T and G-to-T editing with an intron-split EGFP reporter system. (a) Design of the reporter for A-to-T or G-to-T editing detection. P2A, 2A peptide from the porcine teschovirus-1. (b) Percentage of EGFP^+^ cells for evaluation of A editing activity by gABE with various MPG variants. WT: wild-type; v0.2: MPG-N169S; v1: MPG with N169S, S198A, K202A, G203A, S206A and K210A mutations; v2: MPG with mutations N169S and G163R; v3: MPG with G163R, N169S, S198A, K202A, G203A, S206A and K210A mutations (mean ± s.e.m., *n* = 3). (c) Percentage of EGFP^+^ cells representing the efficiency of G-to-T conversion for gGBE variants containing various forms of mutated N-methylpurine DNA glycosylase (MPG). dMPG, inactive dead MPG; T: target sgRNA; NT: non-target sgRNA (mean ± s.e.m., *n* = 3). (d) Representative flow cytometry scatter plots showing gating strategy and the percentages of EGFP^+^ cells for gGBEv3.


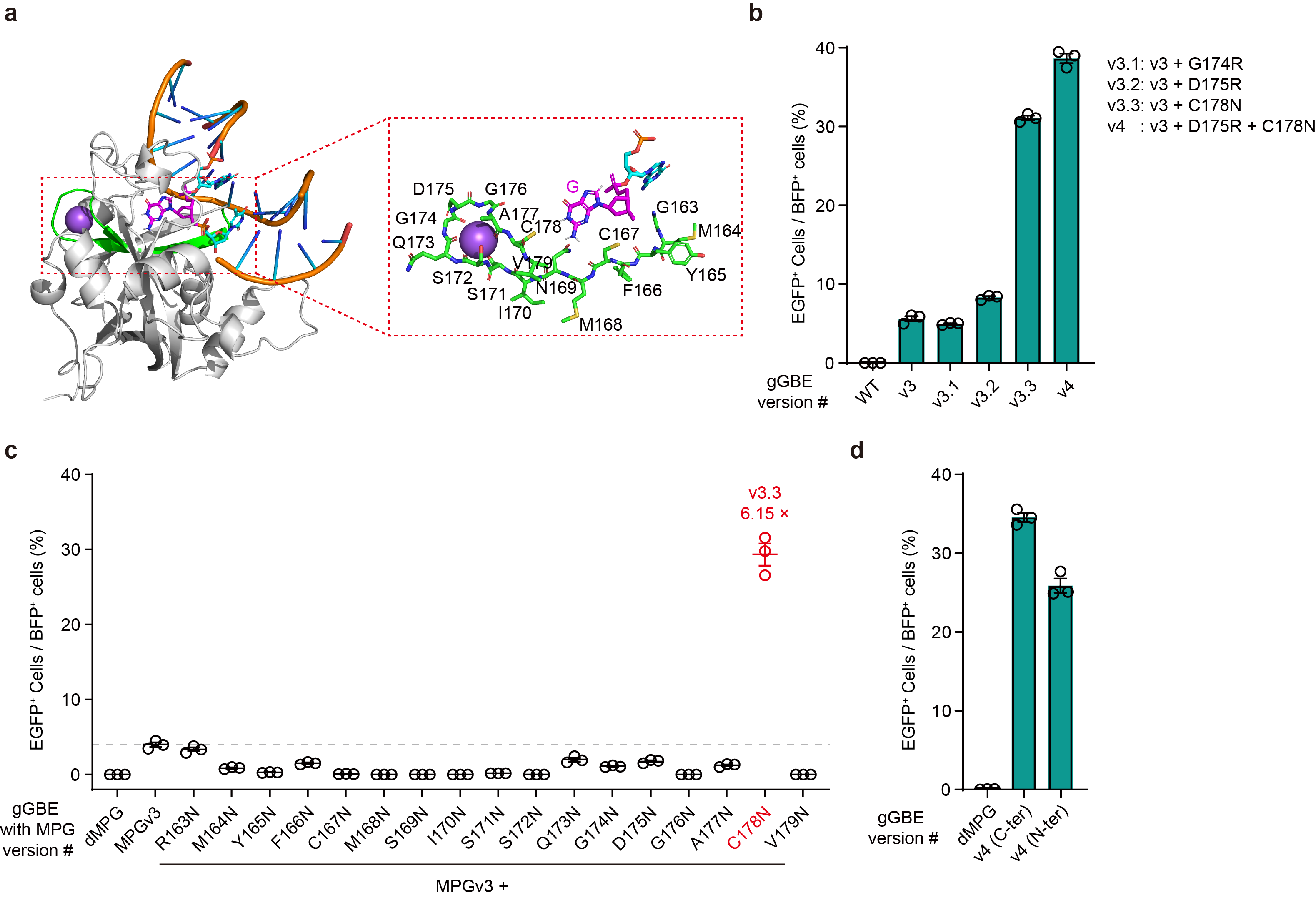


**Supplementary Figure 3.** View of the MPG structure and the first round of mutagenesis. (a) Structures for aa 78-298 region (left) and 163-179 region (right) of human MPG protein (shown in gray), as predicted by AlphaFold (<https://alphafold.com/entry/P29372>) aligned with the crystal structure of MPG (PDB entry 1ewn, not shown), in which εA was mutated to G in the DNA. (b) Percentage of EGFP^+^ cells for evaluating G editing activity with different candidates with various MPG variants (mean ± s.e.m., *n* = 3). (c) Performance of various engineered variants measured by the percentage of EGFP^+^ cells in the first round of screening. Dotted line, mean value of the MPGv3 group. Fold changes were calculated relative to the MPGv3 group (mean ± s.e.m., *n* = 3). (d) Percentage of EGFP^+^ cells for each base editor (mean ± s.e.m., *n* = 3).


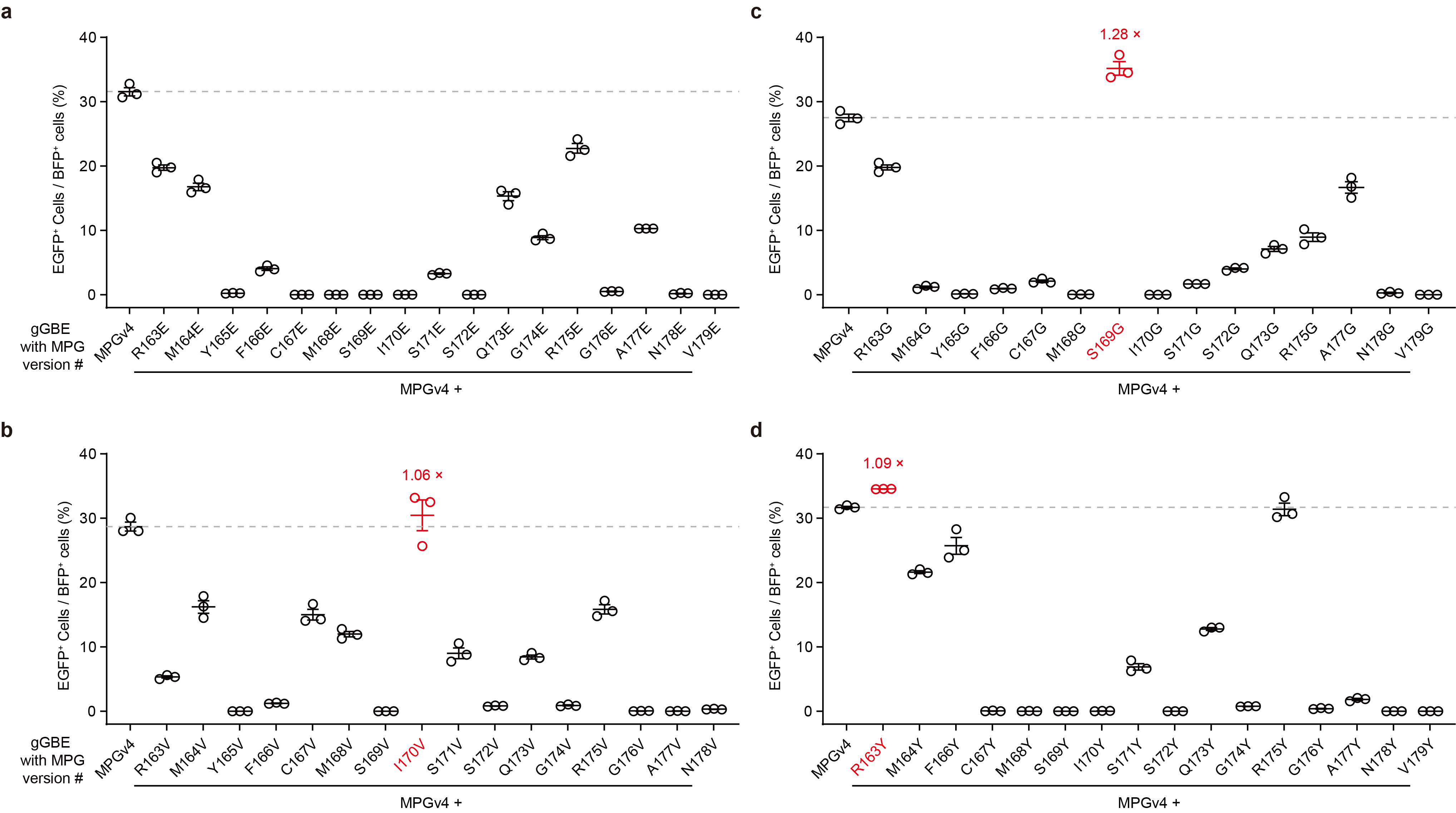


**Supplementary Figure 4.** Performance of engineered variants in the second round of screening. (a-d) Percentage of EGFP^+^ cells of gGBE candidates with different MPG variants from sequential substitutions of glutamic acid (a), valine (b), glycine (c), and tyrosine (d) (X>E, V, G, or Y). *n* = 3. All values are presented as mean ± s.e.m.


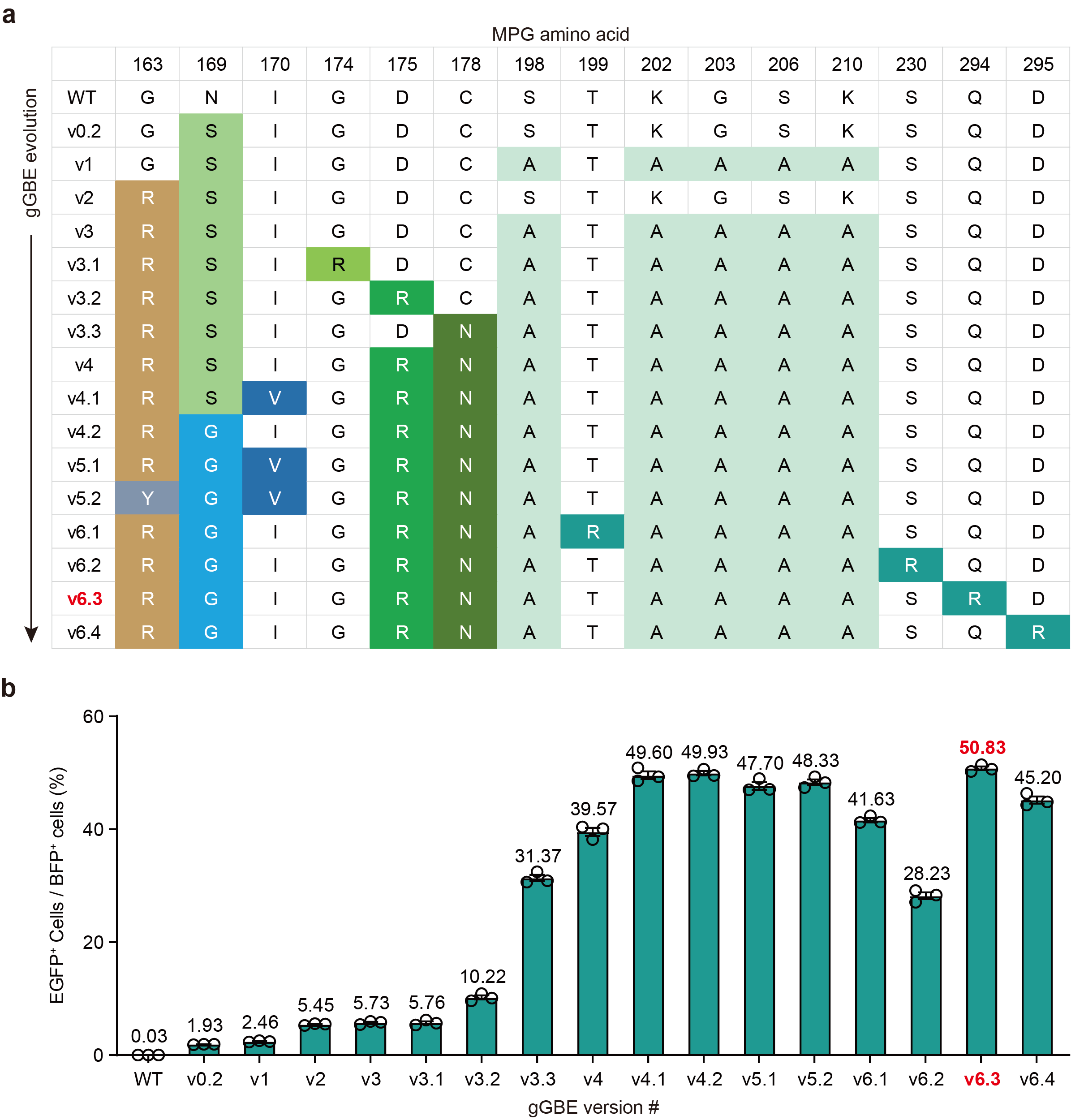


**Supplementary Figure 5.** Progressive engineering and G editing efficiency of gGBEs. (a) Progressive mutations of gGBEs described in this work. Different rounds of mutations are marked with different color shades. (b) Percentage of EGFP^+^ cells for each gGBE variants. *n* = 3. All values are presented as mean ± s.e.m.


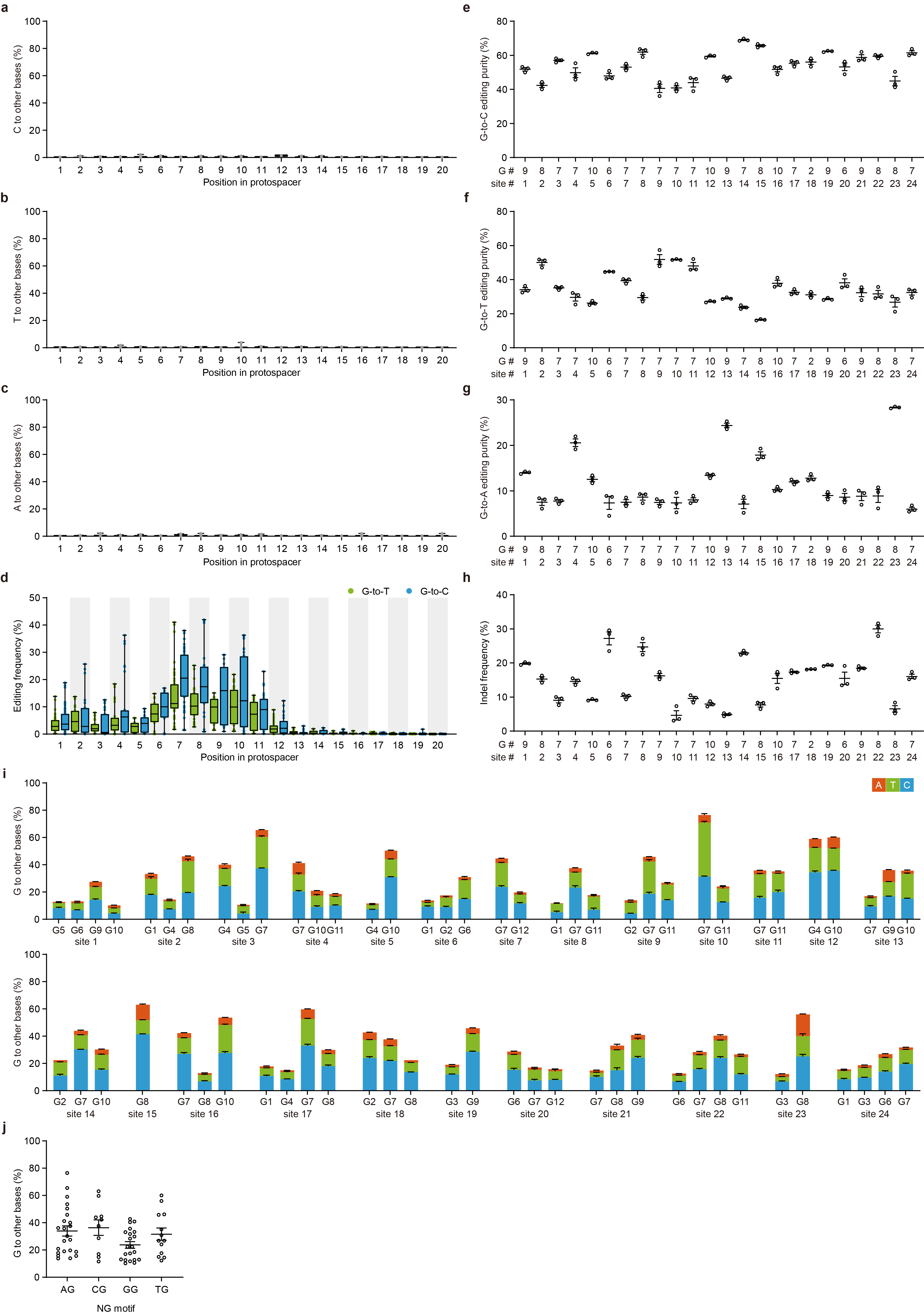


**Supplementary Figure 6.** Further characterization of editing profiles for gGBEv6.3. (a-c) Frequencies of C (a), T (b) and A (c) conversions by gGBEv6.3 across the protospacer positions 1–20 (where PAM is at positions 21–23) from the edited sites in Figure 3a. (d) Frequencies of G-to-T and G-to-C editing by gGBEv6.3. In a-d, single dot represents individual replicate (n = 3 independent replicates per site), and boxes span the interquartile range (25th to 75th percentile); horizontal lines within the boxes indicate the median (50%); and whiskers extend to the minimal and maximal values. (e-g) Percentage of G-to-C (e), G-to-T (f) or G-to-A (g) editing by gGBEv6.3 at various edited sites shown in Figure 3a (mean ± s.e.m., *n* = 3). (h) Indels frequencies with gGBEv6.3 at 24 on-target sites (mean ± s.e.m., *n* = 3). (i) Bar plots showing the on-target DNA base editing at positions with G conversion frequencies >10% at each genomic site in HEK293T cells (mean ± s.e.m., *n* = 3). (j) The statistical analysis of on-target DNA base editing for each NG motif from the edited sites in (i). Each dot represents the mean of three biological replicates for each edited position at various edited sites.

**
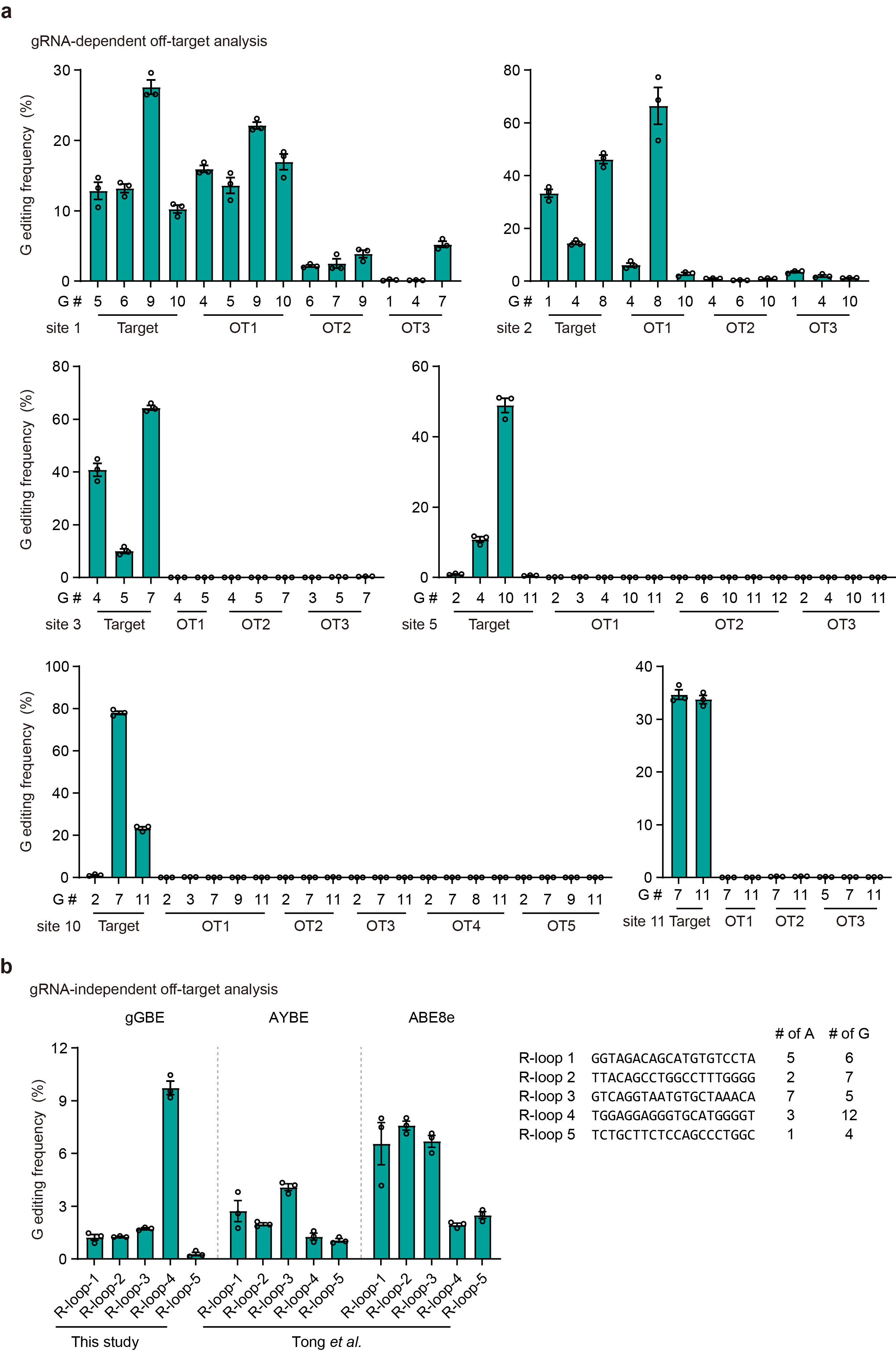
**

**Supplementary Figure 7.** The sgRNA-dependent and sgRNA-independent off-target analysis. (a) The sgRNA-dependent off-target analysis for gGBEv6.3 editing at different sites (*n* = 3). OT: off-target. (b) The sgRNA-independent off-target editing detected by the orthogonal R-loop assay at each R-loop site for gGBE, AYBE and ABE8e (*n* = 3), respectively. Data for AYBE and ABE8e were adopted from Tong *et al.* [1]. All values are presented as mean ± s.e.m.


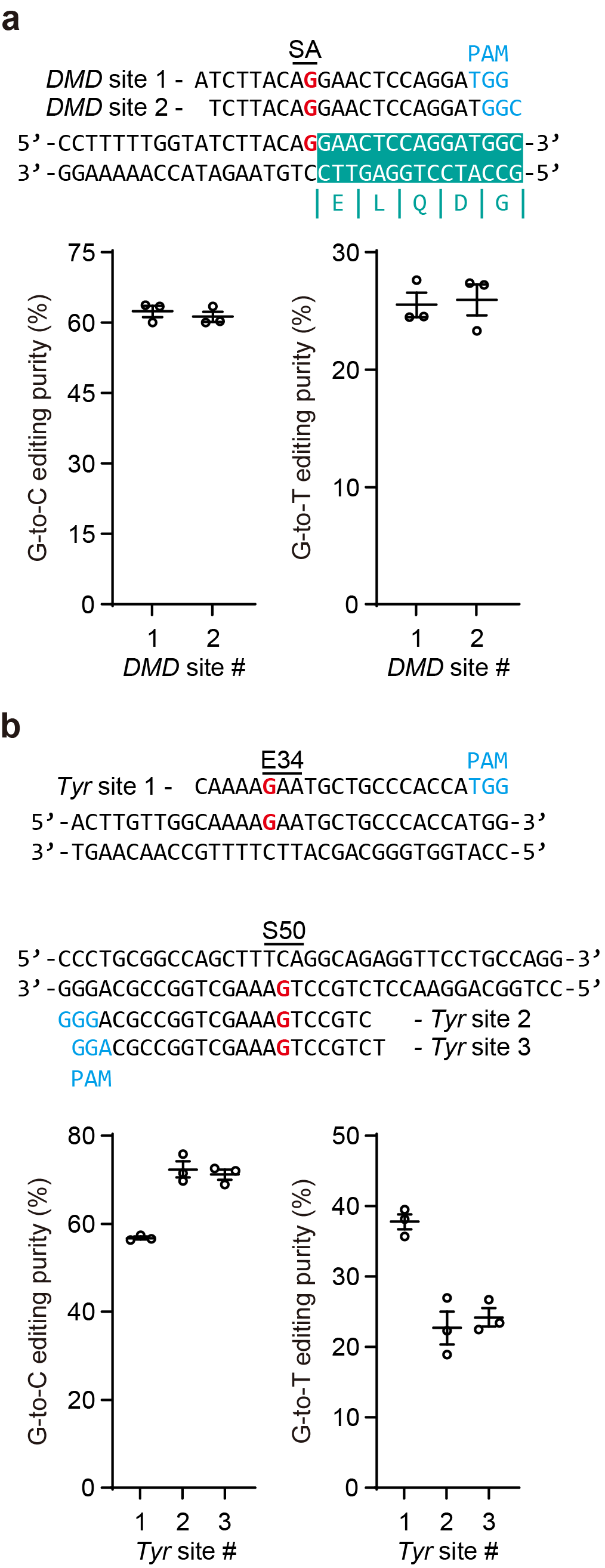


**Supplementary Figure 8.** The percentage of G-to-C and G-to-T among all G-to-C/T/A conversion events at *DMD* or *Tyr* sites targeted by gGBEv6.3. (a) Percentages of G-to-C and G-to-T editing events in HEK293T cells with gGBEv6.3 at two *DMD* sites (corresponding to Fig. 4C). *n* = 3. SA, splicing acceptor site. PAM, Protospacer adjacent motif. (b) Percentages of G-to-C and G-to-T editing events in N2a cells with gGBEv6.3 at three *Tyr* sites (corresponding to Figure 4e). *n* = 3. All values are presented as mean ± s.e.m.


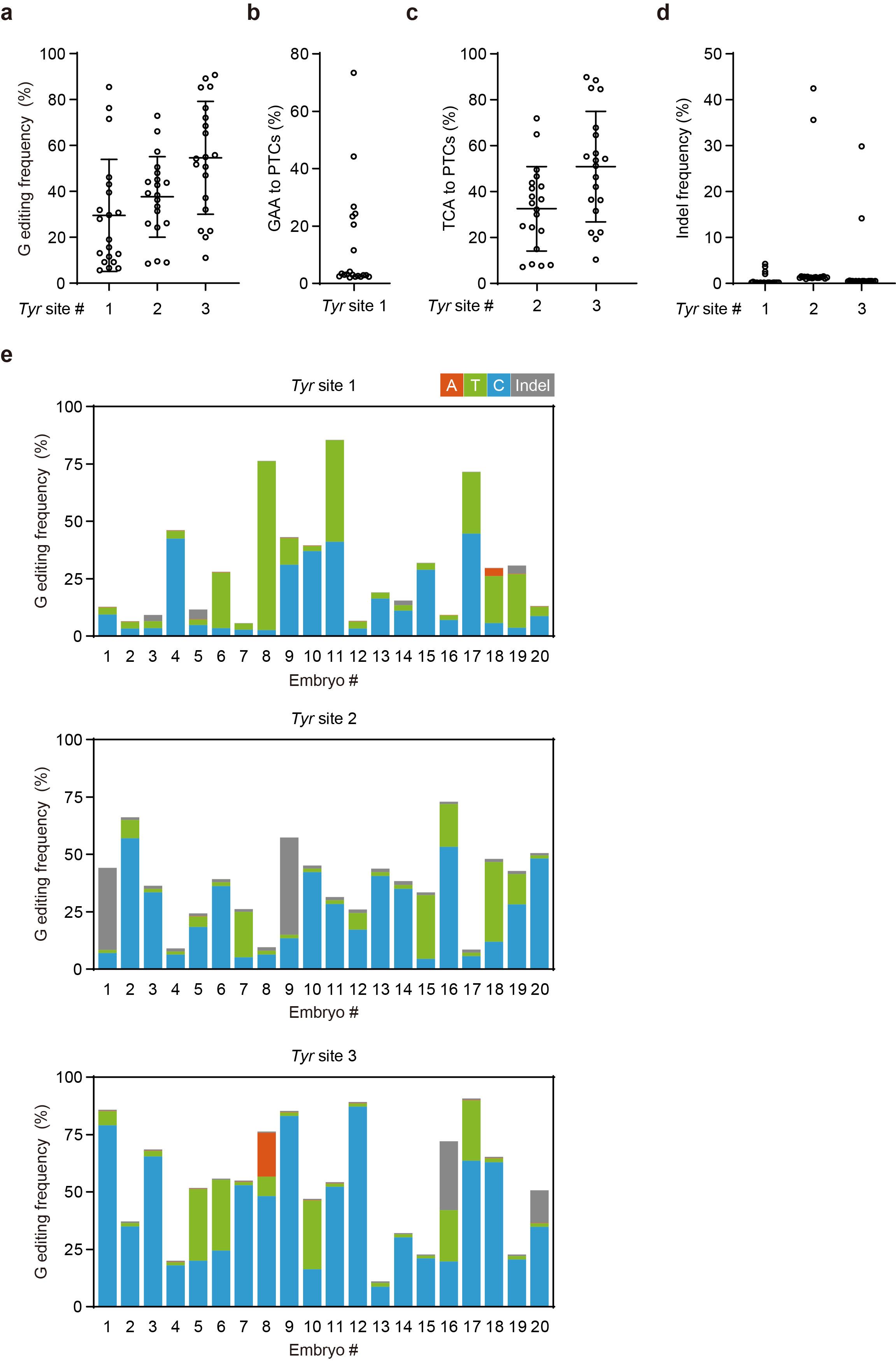


**Supplementary Figure 9.** G editing in mouse embryos with gGBEv6.3. (a) On-target base editing efficiencies for gGBEv6.3 targeting three *Tyr* sites in mouse embryos (mean ± s.e.m., *n* = 20). (b,c) Percentages of G conversion-induced PTCs by gGBEv6.3 at three *Tyr* sites in mouse embryos (mean ± s.e.m., *n* = 20 embryos). (d) Indel frequencies induced by gGBEv6.3 targeting at three *Tyr* sites in mouse embryos shown in **a** (*n* = 20). (e) Bar plots showing the on-target G editing frequencies for individual mouse embryos, with gGBEv6.3 targeting *Tyr* site 1, *Tyr* site 2 and *Tyr* site 3.

**
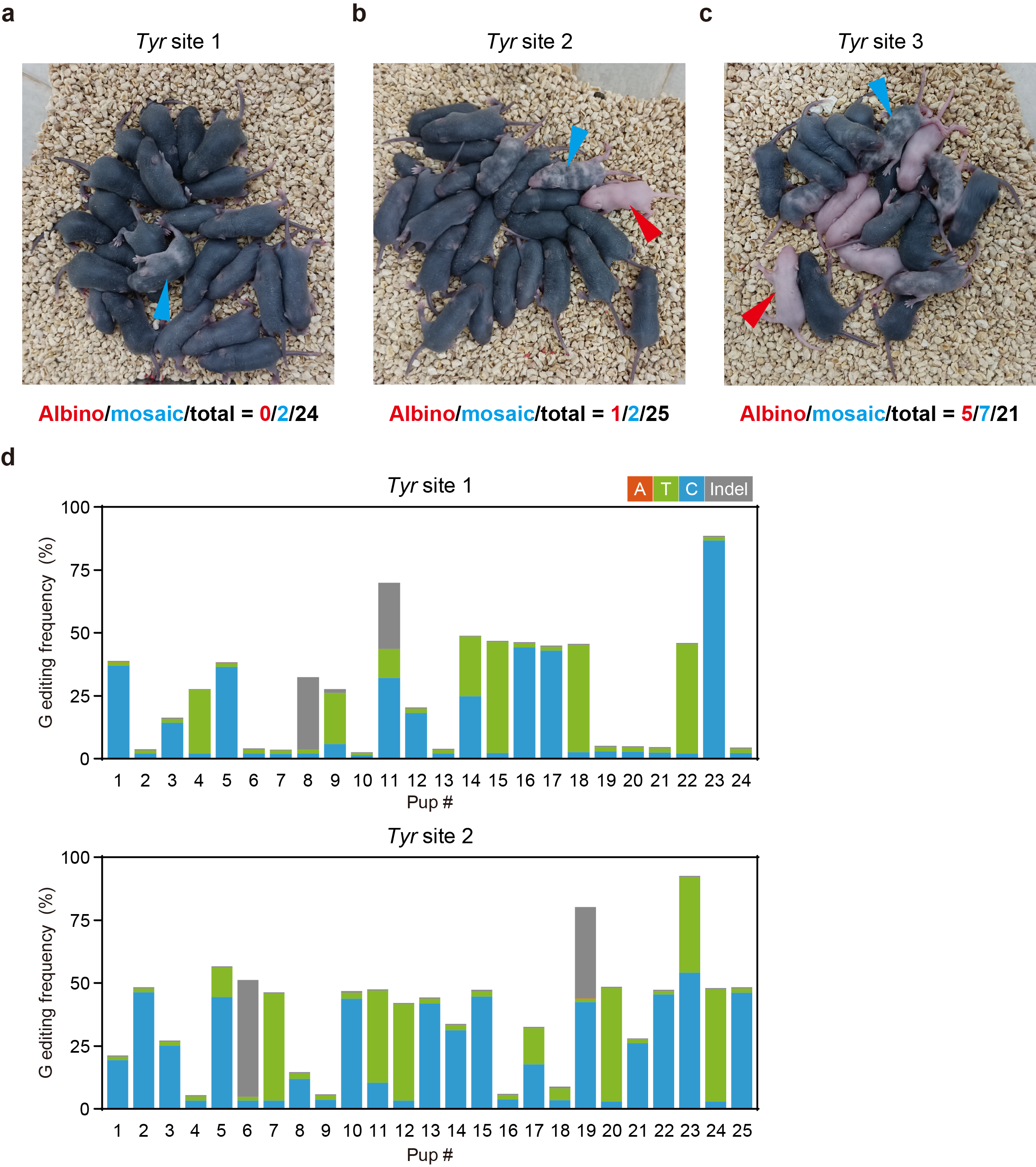
**

**Supplementary Figure 10.** Phenotypes and genotyping of F0 mouse pups. (a-c) Phenotypes of F0 mice generated by microinjection of gGBEv6.3 mRNA and sgRNA for targeting *Tyr* site 1 (a), site 2 (b) and site 3 (c). The images were obtained for P6 mice. Arrowheads: red, albino mice; blue, mice with mosaic pigmentation. (d) Bar plots showing the on-target G editing frequencies for individual mouse pups, with gGBEv6.3 targeting *Tyr* site 1 and *Tyr* site 2.

**SUPPLEMENTARY TABLES**

**Supplementary Table 1. Protein sequence of gGBEv6.3 editor.**

| MPG mutations | Protein sequences (bpNLS; Linker; nCas9(D10A); MPG; mutations) |
| --- | --- |
| G163R,N169G,D175R,C178N,S198A,K202A,G203A,S206A,K210A,Q294R | MKRTADGSEFESPKKKRKVSGGSDKKYSIGLAIGTNSVGWAVITDEYKVPSKKFKVLGNTDRHSIKKNLIGALLFDSGETAEATRLKRTARRRYTRRKNRICYLQEIFSNEMAKVDDSFFHRLEESFLVEEDKKHERHPIFGNIVDEVAYHEKYPTIYHLRKKLVDSTDKADLRLIYLALAHMIKFRGHFLIEGDLNPDNSDVDKLFIQLVQTYNQLFEENPINASGVDAKAILSARLSKSRRLENLIAQLPGEKKNGLFGNLIALSLGLTPNFKSNFDLAEDAKLQLSKDTYDDDLDNLLAQIGDQYADLFLAAKNLSDAILLSDILRVNTEITKAPLSASMIKRYDEHHQDLTLLKALVRQQLPEKYKEIFFDQSKNGYAGYIDGGASQEEFYKFIKPILEKMDGTEELLVKLNREDLLRKQRTFDNGSIPHQIHLGELHAILRRQEDFYPFLKDNREKIEKILTFRIPYYVGPLARGNSRFAWMTRKSEETITPWNFEEVVDKGASAQSFIERMTNFDKNLPNEKVLPKHSLLYEYFTVYNELTKVKYVTEGMRKPAFLSGEQKKAIVDLLFKTNRKVTVKQLKEDYFKKIECFDSVEISGVEDRFNASLGTYHDLLKIIKDKDFLDNEENEDILEDIVLTLTLFEDREMIEERLKTYAHLFDDKVMKQLKRRRYTGWGRLSRKLINGIRDKQSGKTILDFLKSDGFANRNFMQLIHDDSLTFKEDIQKAQVSGQGDSLHEHIANLAGSPAIKKGILQTVKVVDELVKVMGRHKPENIVIEMARENQTTQKGQKNSRERMKRIEEGIKELGSQILKEHPVENTQLQNEKLYLYYLQNGRDMYVDQELDINRLSDYDVDHIVPQSFLKDDSIDNKVLTRSDKNRGKSDNVPSEEVVKKMKNYWRQLLNAKLITQRKFDNLTKAERGGLSELDKAGFIKRQLVETRQITKHVAQILDSRMNTKYDENDKLIREVKVITLKSKLVSDFRKDFQFYKVREINNYHHAHDAYLNAVVGTALIKKYPKLESEFVYGDYKVYDVRKMIAKSEQEIGKATAKYFFYSNIMNFFKTEITLANGEIRKRPLIETNGETGEIVWDKGRDFATVRKVLSMPQVNIVKKTEVQTGGFSKESILPKRNSDKLIARKKDWDPKKYGGFDSPTVAYSVLVVAKVEKGKSKKLKSVKELLGITIMERSSFEKNPIDFLEAKGYKEVKKDLIIKLPKYSLFELENGRKRMLASAGELQKGNELALPSKYVNFLYLASHYEKLKGSPEDNEQKQLFVEQHKHYLDEIIEQISEFSKRVILADANLDKVLSAYNKHRDKPIREQAENIIHLFTLTNLGAPAAFKYFDTTIDRKRYTSTKEVLDATLIHQSITGLYETRIDLSQLGGDSGGSKRTADGSEFEPKKKRKVLGGDSGGSGGSGGSVTPALQMKKPKQFCRRMGQKKQRPARAGQPHSSSDAAQAPAEQPHSSSDAAQAPCPRERCLGPPTTPGPYRSIYFSSPKGHLTRLGLEFFDQPAVPLARAFLGQVLVRRLPNGTELRGRIVETEAYLGPEDEAAHSRGGRQTPRNRGMFMKPGTLYVYIIYRMYFCMGISSQGRGANVLLRALEPLEGLETMRQLRATLRAATAARVLADRELCSGPSKLCQALAINKSFDQRDLAQDEAVWLERGPLEPSEPAVVAAARVGVGHAGEWARKPLRFYVRGSPWVSVVDRVAERDTQASGGSKRTADGSEFEPKKKRKV |

**Supplementary Table 2. MPG variants in rounds of sequential substitutions.**

| Variants | Amino Acid sequence | Nucleotide sequence |
| --- | --- | --- |
| MPGv3-R163N | NMYFCMSISSQGDGACV | aacATGTACTTCTGCATGAGCATCTCCAGCCAGGGGGACGGGGCTTGCGTC |
| MPGv3-M164N | RNYFCMSISSQGDGACV | AGAaacTACTTCTGCATGAGCATCTCCAGCCAGGGGGACGGGGCTTGCGTC |
| MPGv3-Y165N | RMNFCMSISSQGDGACV | AGAATGaacTTCTGCATGAGCATCTCCAGCCAGGGGGACGGGGCTTGCGTC |
| MPGv3-F166N | RMYNCMSISSQGDGACV | AGAATGTACaacTGCATGAGCATCTCCAGCCAGGGGGACGGGGCTTGCGTC |
| MPGv3-C167N | RMYFNMSISSQGDGACV | AGAATGTACTTCaacATGAGCATCTCCAGCCAGGGGGACGGGGCTTGCGTC |
| MPGv3-M168N | RMYFCNSISSQGDGACV | AGAATGTACTTCTGCaacAGCATCTCCAGCCAGGGGGACGGGGCTTGCGTC |
| MPGv3-S169N | RMYFCMNISSQGDGACV | AGAATGTACTTCTGCATGaacATCTCCAGCCAGGGGGACGGGGCTTGCGTC |
| MPGv3-I170N | RMYFCMSNSSQGDGACV | AGAATGTACTTCTGCATGAGCaacTCCAGCCAGGGGGACGGGGCTTGCGTC |
| MPGv3-S171N | RMYFCMSINSQGDGACV | AGAATGTACTTCTGCATGAGCATCaacAGCCAGGGGGACGGGGCTTGCGTC |
| MPGv3-S172N | RMYFCMSISNQGDGACV | AGAATGTACTTCTGCATGAGCATCTCCaacCAGGGGGACGGGGCTTGCGTC |
| MPGv3-Q173N | RMYFCMSISSNGDGACV | AGAATGTACTTCTGCATGAGCATCTCCAGCaacGGGGACGGGGCTTGCGTC |
| MPGv3-G174N | RMYFCMSISSQNDGACV | AGAATGTACTTCTGCATGAGCATCTCCAGCCAGaacGACGGGGCTTGCGTC |
| MPGv3-D175N | RMYFCMSISSQGNGACV | AGAATGTACTTCTGCATGAGCATCTCCAGCCAGGGGaacGGGGCTTGCGTC |
| MPGv3-G176N | RMYFCMSISSQGDNACV | AGAATGTACTTCTGCATGAGCATCTCCAGCCAGGGGGACaacGCTTGCGTC |
| MPGv3-A177N | RMYFCMSISSQGDGNCV | AGAATGTACTTCTGCATGAGCATCTCCAGCCAGGGGGACGGGaacTGCGTC |
| MPGv3-C178N | RMYFCMSISSQGDGANV | AGAATGTACTTCTGCATGAGCATCTCCAGCCAGGGGGACGGGGCTaacGTC |
| MPGv3-V179N | RMYFCMSISSQGDGACN | AGAATGTACTTCTGCATGAGCATCTCCAGCCAGGGGGACGGGGCTTGCaac |
| MPGv4-R163G | GMYFCMSISSQGRGANV | ggcATGTACTTCTGCATGAGCATCTCCAGCCAGGGGAGAGGGGCTAACGTC |
| MPGv4-M164G | RGYFCMSISSQGRGANV | AGAggcTACTTCTGCATGAGCATCTCCAGCCAGGGGAGAGGGGCTAACGTC |
| MPGv4-Y165G | RMGFCMSISSQGRGANV | AGAATGggcTTCTGCATGAGCATCTCCAGCCAGGGGAGAGGGGCTAACGTC |
| MPGv4-F166G | RMYGCMSISSQGRGANV | AGAATGTACggcTGCATGAGCATCTCCAGCCAGGGGAGAGGGGCTAACGTC |
| MPGv4-C167G | RMYFGMSISSQGRGANV | AGAATGTACTTCggcATGAGCATCTCCAGCCAGGGGAGAGGGGCTAACGTC |
| MPGv4-M168G | RMYFCGSISSQGRGANV | AGAATGTACTTCTGCggcAGCATCTCCAGCCAGGGGAGAGGGGCTAACGTC |
| MPGv4-S169G | RMYFCMGISSQGRGANV | AGAATGTACTTCTGCATGggcATCTCCAGCCAGGGGAGAGGGGCTAACGTC |
| MPGv4-I170G | RMYFCMSGSSQGRGANV | AGAATGTACTTCTGCATGAGCggcTCCAGCCAGGGGAGAGGGGCTAACGTC |
| MPGv4-S171G | RMYFCMSIGSQGRGANV | AGAATGTACTTCTGCATGAGCATCggcAGCCAGGGGAGAGGGGCTAACGTC |
| MPGv4-S172G | RMYFCMSISGQGRGANV | AGAATGTACTTCTGCATGAGCATCTCCggcCAGGGGAGAGGGGCTAACGTC |
| MPGv4-Q173G | RMYFCMSISSGGRGANV | AGAATGTACTTCTGCATGAGCATCTCCAGCggcGGGAGAGGGGCTAACGTC |
| MPGv4-R175G | RMYFCMSISSQGGGANV | AGAATGTACTTCTGCATGAGCATCTCCAGCCAGGGGggcGGGGCTAACGTC |
| MPGv4-A177G | RMYFCMSISSQGRGGNV | AGAATGTACTTCTGCATGAGCATCTCCAGCCAGGGGAGAGGGggcAACGTC |
| MPGv4-N178G | RMYFCMSISSQGRGAGV | AGAATGTACTTCTGCATGAGCATCTCCAGCCAGGGGAGAGGGGCTggcGTC |
| MPGv4-V179G | RMYFCMSISSQGRGANG | AGAATGTACTTCTGCATGAGCATCTCCAGCCAGGGGAGAGGGGCTAACggc |
| MPGv4-R163E | EMYFCMSISSQGRGANV | gagATGTACTTCTGCATGAGCATCTCCAGCCAGGGGAGAGGGGCTAACGTC |
| MPGv4-M164E | REYFCMSISSQGRGANV | AGAgagTACTTCTGCATGAGCATCTCCAGCCAGGGGAGAGGGGCTAACGTC |
| MPGv4-Y165E | RMEFCMSISSQGRGANV | AGAATGgagTTCTGCATGAGCATCTCCAGCCAGGGGAGAGGGGCTAACGTC |
| MPGv4-F166E | RMYECMSISSQGRGANV | AGAATGTACgagTGCATGAGCATCTCCAGCCAGGGGAGAGGGGCTAACGTC |
| MPGv4-C167E | RMYFEMSISSQGRGANV | AGAATGTACTTCgagATGAGCATCTCCAGCCAGGGGAGAGGGGCTAACGTC |
| MPGv4-M168E | RMYFCESISSQGRGANV | AGAATGTACTTCTGCgagAGCATCTCCAGCCAGGGGAGAGGGGCTAACGTC |
| MPGv4-S169E | RMYFCMEISSQGRGANV | AGAATGTACTTCTGCATGgagATCTCCAGCCAGGGGAGAGGGGCTAACGTC |
| MPGv4-I170E | RMYFCMSESSQGRGANV | AGAATGTACTTCTGCATGAGCgagTCCAGCCAGGGGAGAGGGGCTAACGTC |
| MPGv4-S171E | RMYFCMSIESQGRGANV | AGAATGTACTTCTGCATGAGCATCgagAGCCAGGGGAGAGGGGCTAACGTC |
| MPGv4-S172E | RMYFCMSISEQGRGANV | AGAATGTACTTCTGCATGAGCATCTCCgagCAGGGGAGAGGGGCTAACGTC |
| MPGv4-Q173E | RMYFCMSISSEGRGANV | AGAATGTACTTCTGCATGAGCATCTCCAGCgagGGGAGAGGGGCTAACGTC |
| MPGv4-G174E | RMYFCMSISSQERGANV | AGAATGTACTTCTGCATGAGCATCTCCAGCCAGgagAGAGGGGCTAACGTC |
| MPGv4-R175E | RMYFCMSISSQGEGANV | AGAATGTACTTCTGCATGAGCATCTCCAGCCAGGGGgagGGGGCTAACGTC |
| MPGv4-G176E | RMYFCMSISSQGREANV | AGAATGTACTTCTGCATGAGCATCTCCAGCCAGGGGAGAgagGCTAACGTC |
| MPGv4-A177E | RMYFCMSISSQGRGENV | AGAATGTACTTCTGCATGAGCATCTCCAGCCAGGGGAGAGGGgagAACGTC |
| MPGv4-N178E | RMYFCMSISSQGRGAEV | AGAATGTACTTCTGCATGAGCATCTCCAGCCAGGGGAGAGGGGCTgagGTC |
| MPGv4-V179E | RMYFCMSISSQGRGANE | AGAATGTACTTCTGCATGAGCATCTCCAGCCAGGGGAGAGGGGCTAACgag |
| MPGv4-R163V | VMYFCMSISSQGRGANV | gtgATGTACTTCTGCATGAGCATCTCCAGCCAGGGGAGAGGGGCTAACGTC |
| MPGv4-M164V | RVYFCMSISSQGRGANV | AGAgtgTACTTCTGCATGAGCATCTCCAGCCAGGGGAGAGGGGCTAACGTC |
| MPGv4-Y165V | RMVFCMSISSQGRGANV | AGAATGgtgTTCTGCATGAGCATCTCCAGCCAGGGGAGAGGGGCTAACGTC |
| MPGv4-F166V | RMYVCMSISSQGRGANV | AGAATGTACgtgTGCATGAGCATCTCCAGCCAGGGGAGAGGGGCTAACGTC |
| MPGv4-C167V | RMYFVMSISSQGRGANV | AGAATGTACTTCgtgATGAGCATCTCCAGCCAGGGGAGAGGGGCTAACGTC |
| MPGv4-M168V | RMYFCVSISSQGRGANV | AGAATGTACTTCTGCgtgAGCATCTCCAGCCAGGGGAGAGGGGCTAACGTC |
| MPGv4-S169V | RMYFCMVISSQGRGANV | AGAATGTACTTCTGCATGgtgATCTCCAGCCAGGGGAGAGGGGCTAACGTC |
| MPGv4-I170V | RMYFCMSVSSQGRGANV | AGAATGTACTTCTGCATGAGCgtgTCCAGCCAGGGGAGAGGGGCTAACGTC |
| MPGv4-S171V | RMYFCMSIVSQGRGANV | AGAATGTACTTCTGCATGAGCATCgtgAGCCAGGGGAGAGGGGCTAACGTC |
| MPGv4-S172V | RMYFCMSISVQGRGANV | AGAATGTACTTCTGCATGAGCATCTCCgtgCAGGGGAGAGGGGCTAACGTC |
| MPGv4-Q173V | RMYFCMSISSVGRGANV | AGAATGTACTTCTGCATGAGCATCTCCAGCgtgGGGAGAGGGGCTAACGTC |
| MPGv4-G174V | RMYFCMSISSQVRGANV | AGAATGTACTTCTGCATGAGCATCTCCAGCCAGgtgAGAGGGGCTAACGTC |
| MPGv4-R175V | RMYFCMSISSQGVGANV | AGAATGTACTTCTGCATGAGCATCTCCAGCCAGGGGgtgGGGGCTAACGTC |
| MPGv4-G176V | RMYFCMSISSQGRVANV | AGAATGTACTTCTGCATGAGCATCTCCAGCCAGGGGAGAgtgGCTAACGTC |
| MPGv4-A177V | RMYFCMSISSQGRGVNV | AGAATGTACTTCTGCATGAGCATCTCCAGCCAGGGGAGAGGGgtgAACGTC |
| MPGv4-N178V | RMYFCMSISSQGRGAVV | AGAATGTACTTCTGCATGAGCATCTCCAGCCAGGGGAGAGGGGCTgtgGTC |
| MPGv4-R163Y | YMYFCMSISSQGRGANV | tacATGTACTTCTGCATGAGCATCTCCAGCCAGGGGAGAGGGGCTAACGTC |
| MPGv4-M164Y | RYYFCMSISSQGRGANV | AGAtacTACTTCTGCATGAGCATCTCCAGCCAGGGGAGAGGGGCTAACGTC |
| MPGv4-F166Y | RMYYCMSISSQGRGANV | AGAATGTACtacTGCATGAGCATCTCCAGCCAGGGGAGAGGGGCTAACGTC |
| MPGv4-C167Y | RMYFYMSISSQGRGANV | AGAATGTACTTCtacATGAGCATCTCCAGCCAGGGGAGAGGGGCTAACGTC |
| MPGv4-M168Y | RMYFCYSISSQGRGANV | AGAATGTACTTCTGCtacAGCATCTCCAGCCAGGGGAGAGGGGCTAACGTC |
| MPGv4-S169Y | RMYFCMYISSQGRGANV | AGAATGTACTTCTGCATGtacATCTCCAGCCAGGGGAGAGGGGCTAACGTC |
| MPGv4-I170Y | RMYFCMSYSSQGRGANV | AGAATGTACTTCTGCATGAGCtacTCCAGCCAGGGGAGAGGGGCTAACGTC |
| MPGv4-S171Y | RMYFCMSIYSQGRGANV | AGAATGTACTTCTGCATGAGCATCtacAGCCAGGGGAGAGGGGCTAACGTC |
| MPGv4-S172Y | RMYFCMSISYQGRGANV | AGAATGTACTTCTGCATGAGCATCTCCtacCAGGGGAGAGGGGCTAACGTC |
| MPGv4-Q173Y | RMYFCMSISSYGRGANV | AGAATGTACTTCTGCATGAGCATCTCCAGCtacGGGAGAGGGGCTAACGTC |
| MPGv4-G174Y | RMYFCMSISSQYRGANV | AGAATGTACTTCTGCATGAGCATCTCCAGCCAGtacAGAGGGGCTAACGTC |
| MPGv4-R175Y | RMYFCMSISSQGYGANV | AGAATGTACTTCTGCATGAGCATCTCCAGCCAGGGGtacGGGGCTAACGTC |
| MPGv4-G176Y | RMYFCMSISSQGRYANV | AGAATGTACTTCTGCATGAGCATCTCCAGCCAGGGGAGAtacGCTAACGTC |
| MPGv4-A177Y | RMYFCMSISSQGRGYNV | AGAATGTACTTCTGCATGAGCATCTCCAGCCAGGGGAGAGGGtacAACGTC |
| MPGv4-N178Y | RMYFCMSISSQGRGAYV | AGAATGTACTTCTGCATGAGCATCTCCAGCCAGGGGAGAGGGGCTtacGTC |
| MPGv4-V179Y | RMYFCMSISSQGRGANY | AGAATGTACTTCTGCATGAGCATCTCCAGCCAGGGGAGAGGGGCTAACtac |

**Supplementary Table 3. Sequences of sgRNAs and primers for genomic DNA targeted amplification used in this study.**

| On-target |  |  |  |  |  |  |
| --- | --- | --- | --- | --- | --- | --- |
| site # | Gene | Spacer sequence | PAM | Source (PMID) | Forword-primer | Reverse-primer |
| site 1 | *HBG* | GTGGGGAAGGGGCCCCCAAG | AGG | 32433547 | CTGGCCTCACTGGATACTC | CTACAGGCCTCACTGGAGC |
| site 2 | *VEGFA* | GGTGAGTGAGTGTGTGCGTG | TGG | 32433547 | CCCATTCCCTCTTTAGCCAG | GTGAGGTTACGTGCGGACAG |
| site 3 | *PCSK9* | CTAGGAGATACACCTCCACC | AGG | 36624150 | GAGTACAGCTGCAACGCTC | GAGCAGGATGACTTGGGTCC |
| site 4 | *FANCF* | GCTGCAGAAGGGATTCCATG | AGG | 32690971 | CTCTTGCCTCCACTGGTTG | GAAGAACCTCTTTGTGTGGCG |
| site 5 | *FANCF* | AGCGATCCAGGTGCTGCAGA | AGG | 32690971 | CTCTTGCCTCCACTGGTTG | GAAGAACCTCTTTGTGTGGCG |
| site 6 | *LINC01509* | GGATTGACCCAGGCCAGGGC | TGG | 29160308 | GGTCCCTCCTCTCCTGGTGA | TCACAGTGGCAAATGAGGCT |
| site 7 | *EMX1* | GAGTCCGAGCAGAAGAAGAA | GGG | 32433547 | CACTGTGTCCTCTTCCTGCC | CCCATTGCTTGTCCCTCTGTC |
| site 8 |  | GAGCCAGAATGAGCACGTGA | GGG | 32042165 | AACTCTTGCTCTTTGGGGCC | CACTTAGGAGCTGCAGGCTTT |
| site 9 | *PLS3-AS1* | AGACCAGACTGAGCAAGAGA | GGG | 32042165 | CTGGTCTCAAATTCCCGACTTGG | GGCTTGCTTCATGAAACCGAC |
| site 10 | *MKNK1-AS1* | AGCTCAGACTGAGCAAGTGA | GGG | 32042165 | CAGGCCCATGGATTGGCTAG | CTGAAGTCCCTTCAGGTCAGC |
| site 11 | *MINAR1* | CACCCAGACTGAGCACGTGC | TGG | 32042165 | CTATCCAAATGCCAGTAGCCAG | GGGAACTGTCGCAGTCTGAC |
| site 12 | *HBB* | TCAGAAAGTGGTGGCTGGTG | TGG | 29702637 | ATCATGCCTCTTTGCACCATTC | TGCACTGACCTCCCACATTC |
| site 13 | *TCEA3* | AAGTCCGAGGAGAGGAAGAA | AGG | 32042165 | GGAAACTCAGGCGTGTAGAGG | TATAAGCCCTGCGGAGATGGC |
| site 14 | *GJB2* | GGACACGAAGATCAGCTGCA | GGG | 33654077 | GAAGCCGTCGTACATGACATAG | CCGCCCAGAGTAGAAGATGG |
| site 15 | *VISTA enhancer hs267* | CCAGCCCGCTGGCCCTGTAA | AGG | 33654077 | AGAGGTCCTAAACCAGTGTCAG | CTGGTGGTACTTGAATCAAGCAC |
| site 16 | *FGF17* | GGTGGCGGAGATGCGACGCC | CGG | 34211162 | GGAGAAAGACCTCCGTCCATC | TATTCACCTCCTCACCCCTCTC |
| site 17 | *CHM* | GATGGCGGATACTCTCCCTT | CGG | 34211162 | TCAACCCTCCAGGCTAAATGAG | CCTACTCAAATGGCGATAAGCAC |
| site 18 |  | GGAAACGGATAGTTCTGAAA | GGG | 33654077 | TACTGCAGTGGGCCAGATAGAG | CAGTTCTGGGGAATATTTCCGTG |
| site 19 |  | GAGGTCGTGGCTGAGCACAA | GGG | 33654077 | GTAATAGCTGGTTGGCTGAGC | GCAACCTGAAGCACAGGAAC |
| site 20 | *DPYSL3* | GAGCCGGAGCAGAAGAAGGA | GGG | 32042165 | CGCAGATCAGCCATTCTTGTC | TGTTTTCCCCAAACCCCAGG |
| site 21 |  | GAGTCCGGGAAGGAGAAGAA | AGG | 32042165 | GGCTGACTCATCTTCCCCAAAG | GAGACGACCATCTTCTGGCG |
| site 22 | *SBK1* | GTCAGGGGAAGAGCACGTGA | CGG | 34385461 | GACAATGCTGATGCAGTATGGAC | AACCAGAGGCTGAAGAGCAG |
| site 23 | *PPP1R12C* | CTGACCTGCATTCTCTCCCC | TGG | 34385461 | CCAGCTCCCATAGCTCAGTC | TTGGGTGAGGGAGGAGAGATG |
| site 24 | *KCNMA1* | GAGATGGAAGCAGCCAGGGA | TGG | 34012094 | AGATCTGTGCACTTCAGGTGG | CCAACAGCTGCTTCTTTCTCATC |
| *Tyr*-site 1 | *mouse Tyr* | CAAAAGAATGCTGCCCACCA | TGG | This study | CAGGCTGAGAGTATTTGATGTAAGAAGG | ATGGGTGTTGACCCATTGTTC |
| *Tyr*-site 2 | *mouse Tyr* | CTGCCTGAAAGCTGGCCGCA | GGG | This study | CAGGCTGAGAGTATTTGATGTAAGAAGG | ATGGGTGTTGACCCATTGTTC |
| *Tyr*-site 3 | *mouse Tyr* | TCTGCCTGAAAGCTGGCCGC | AGG | This study | CAGGCTGAGAGTATTTGATGTAAGAAGG | ATGGGTGTTGACCCATTGTTC |
| *DMD*-site 1 | *DMD* | ATCTTACAGGAACTCCAGGA | TGG | This study | TGGAGCTAACCGAGAGGGTG | GCCTTTCACCCTGCTTATAATCTC |
| *DMD*-site 2 | *DMD* | TCTTACAGGAACTCCAGGAT | GGC | This study | TGGAGCTAACCGAGAGGGTG | GCCTTTCACCCTGCTTATAATCTC |
|  |  |  |  |  |  |  |
| Off-target |  |  |  |  |  |  |
| site 1-OT1 | *LINC00620* | GGTGGGATGGGGTCCCCAAG | TGG | 32433547 | TCCCTGCAAGCTGAAGAAGG | ACCTCTGAACCAGAGCCTG |
| site 1-OT2 |  | GGTAGGGAGAGGCCCCCAGA | GGG | 32433547 | CAGGGAGAGGGATTGTGGAC | CTGTCCTGGTCCCTACTGAC |
| site 1-OT3 |  | GGTGGGGAGCGGCCCCCCAG | TGG | 32433547 | CGCAACATCGCACAGTGAC | CCAACTCCTGGCTTCAAGTG |
| site 2-OT1 | *MAX* | GAGTGAGTGAGTGTGTGTGTG | GGG | 32433547 | TCTCCTGACAACTCGCGGTC | TCACGTCGCTCTCCACCTC |
| site 2-OT2 | *LINC03033* | GTGTGAGTAAGTGTGTGTGTG | TGG | 32433547 | TGGACGGTCCTGACAGAGAC | GCACATACTGCAGCACCTG |
| site 2-OT3 |  | GGTGAGTGTGTGTGTGCATG | TGG | 32433547 | GCCAGTACAATTGATAGCACCAG | GTTCCCATGGACATTGGCTATG |
| site 7-OT1 | *HCN1* | GAGTTAGAGCAGAAGAAGAA | AGG | 32433547 | GCCCCTCTAATACAATGGGA | AGATTTGCATCTGTGGAGGC |
| site 7-OT2 | *MFAP1* | GAGTCTAAGCAGAAGAAGAA | GAG | 32433547 | TCATCTGAGGACTCCATAGG | GTTGAGGTGGGATACCATGA |
| site 7-OT3 |  | GAGGCCGAGCAGAAGAAAGA | CGG | 32433547 | GCAGTCCTCAGTCTGGCCAG | TTATCCCAATACGCACGCGC |
| site 3-OT1 |  | CCAGGATATACACCACCACC | AGG | Cas-OFFinder | ACAGTGTTAGCATTCCTCTCCC | GGGAGCTCATCCTTACTTGAGG |
| site 3-OT2 |  | CTAGGAGATAAAATTCCACC | GGG | Cas-OFFinder | GGTGGAGACTCTTCCTCATGC | AAATTGATGCCCTTGGTGAGC |
| site 3-OT3 |  | CTGAGAGATACACCTCCACG | TGG | Cas-OFFinder | GAATTGAATGGCCTGAGAACAGTC | GTCCAAAGTCCTCTTCATGACCC |
| site 5-OT1 |  | AGGGATCCAGGTGCTGAAAA | GGG | Cas-OFFinder | GGAGCAGGAGCAGAAATGGTC | TGGGGCATGAACACAGTTCTC |
| site 5-OT2 |  | AGCTAGCCAGGGGCTGCAGA | GGG | Cas-OFFinder | TTCACGAGAGGTTTCTGCAGG | CCAGAGTAGACCCAGCTGAG |
| site 5-OT3 |  | AGCGATCCAGGTGCAGCTCA | AGG | Cas-OFFinder | AATACAACGAGGCAGCCAAC | CAGCATCTTAGGAATTCAGAGGTG |
| site 10-OT1 |  | AGGTCAGAGTGAGCAAGTGA | GGG | Cas-OFFinder | AAGTTGGTCAGGGAAGGCAG | ACATCCCATCTGCAGTAGGTG |
| site 10-OT2 |  | AGCTCAGAATGAGCAAGAGA | TGG | Cas-OFFinder | TCCTGCAACTACAAGAGTGGC | CAGTAGCATCATAGGGATTAGGTTC |
| site 10-OT3 |  | AGCACAGACTGAGGCAGTGA | GGG | Cas-OFFinder | TTCTCTGGGCAGAAACAGGG | GTCTGTCCCAGGACTCTCCT |
| site 10-OT4 |  | AGCTCAGGCAGAGCAAGAGA | GGG | Cas-OFFinder | CAGGACTAGAAGGCATTTGACAC | GATTCCCGACCTCTTCTCGC |
| site 10-OT5 |  | AGCTCAGAGAGAGCAAGGGA | GGG | Cas-OFFinder | GTATGGCCAGGCCTTATAGCC | GTGGCTTTGAGGGCAGAGAG |
| site 11-OT1 |  | CACCCAGACAGAGCACCTGT | GGG | Cas-OFFinder | GAGCAGCAGGAGGAATGATAG | AGTTCAGAGACTCTGGGAGACC |
| site 11-OT2 |  | CACCAAGACAGAACACGTGC | TGG | Cas-OFFinder | TCCACACCAGCTGTCCAATTC | GTGTTTAAGCGGACATGCAC |
| site 11-OT3 |  | CACCGAGACTGACCATGTGC | TGG | Cas-OFFinder | AGTGCCCCTTAATCATTCATTGC | AAGGTGATAGATACTCCAATGACACTG |
| R-loop 1 | *EMX1* | GTGGTAGACAGCATGTGTCCTA | AAGGGT | 32433547 | CTGTGTGGTGGAGTGCTCTG | GCATCACCCTTGTCTTTGCAG |
| R-loop 2 | *GAPDH* | ATTTACAGCCTGGCCTTTGGGG | TCGGGT | 32433547 | GTGGGAGCACAGGTAAGTGC | AGGTCCTCTTGTGTCCCCTC |
| R-loop 3 | *SSH2* | GTGTCAGGTAATGTGCTAAACA | GAGAGT | 32433547 | AGGAGTAGGAGAGGGAGCTG | TCTCCTGACCTCGTGATCCAC |
| R-loop 4 | *LINC02818* | GGTGGAGGAGGGTGCATGGGGT | CAGAAT | 32433547 | TAGGGCATGCCAGATACCAG | GCATACACTCCTGGCATCGC |
| R-loop 5 | *LINC01509* | TCTGCTTCTCCAGCCCTGGC | CTGGGT | 32433547 | TGCAGGGAGCTTGGCATGAG | CAGGGACCTCCCTAGGTGCT |

**REFERENCES**

1. Tong H, Wang X, Liu Y, et al.; Programmable A-to-Y base editing by fusing an adenine base editor with an N-methylpurine DNA glycosylase. *Nat Biotechnol* 2023. doi: 10.1038/s41587-022-01595-6.

2. Neugebauer ME, Hsu A, Arbab M, et al.; Evolution of an adenine base editor into a small, efficient cytosine base editor with low off-target activity. *Nat Biotechnol* 2022. doi: 10.1038/s41587-022-01533-6.
